# Identification and characterization of serine protease inhibitors in a parasitic wasp, *Pteromalus puparum*

**DOI:** 10.1038/s41598-017-16000-5

**Published:** 2017-11-16

**Authors:** Lei Yang, Yaotian Mei, Qi Fang, Jiale Wang, Zhichao Yan, Qisheng Song, Zhe Lin, Gongyin Ye

**Affiliations:** 1 State Key Laboratory of Rice Biology & Ministry of Agriculture Key Lab of Molecular Biology of Crop Pathogens and Insects, Institute of Insect Sciences, Zhejiang University, Hangzhou, 310058 China; 2 Division of Plant Sciences, College of Agriculture, Food and Natural Resources, University of Missouri, Columbia, Missouri USA; 3 State Key Laboratory of Integrated Management of Pest Insects and Rodents, Institute of Zoology, Chinese Academy of Sciences, Beijing, 100101 China

## Abstract

Serine protease inhibitors (SPIs) regulate protease-mediated activities by inactivating their cognate proteinases, and are involved in multiple physiological processes. SPIs have been extensively studied in vertebrates and invertebrates; however, little SPI information is available in parasitoids. Herein, we identified 57 SPI genes in total through the genome of a parasitoid wasp, *Pteromalus puparum*. Gene structure analyses revealed that these SPIs contain 7 SPI domains. Depending on their mode of action, these SPIs can be categorized into serpins, canonical inhibitors and alpha-2-macroglobulins (A2Ms). For serpins and canonical inhibitors, we predicted their putative inhibitory activities to trypsin/chymotrypsin/elastase-like enzymes based on the amino acids in cleaved reactive sites. Sequence alignment and phylogenetic tree indicated that some serpins similar to known functional inhibitory serpins may participate in immune responses. Transcriptome analysis also showed some canonical SPI genes displayed distinct expression patterns in the venom gland and this was confirmed by quantitative real-time PCR (qPCR) analysis, suggesting their specific physiological functions as venom proteins in suppressing host immune responses. The study provides valuable information to clarify the functions of SPIs in digestion, development, reproduction and innate immunity.

## Introduction

Serine proteases (SPs), account for almost one-third of all proteases, and serve as inevitable components in catalyzing hydrolytic reactions both intra- and extracellularly. SPs participate in various physiological processes such as food digestion, embryo development and immune defense^1^. Although SPs are of great importance, sometimes they may be potentially hazardous to their enzymatic environments when not being properly controlled^2^. The hazard of excessive peptidase activities includes inappropriate coagulation, melanization, tissue damage, etc.^3,4^. In fact, the action of SPs is tightly regulated by their inhibitors, including serine protease inhibitors (SPIs)^5–7^. When SPs finish their work, they are inactivated by SPIs and then move out of the circulation, to keep the homeostasis of living body. The roles of SPIs include blood coagulation, reproduction, complement system and innate immune response^8,9^.

Based on the mechanisms of action, SPIs can be classified into serpins, α-macroglobulins (A2Ms), canonical inhibitors and non-canonical inhibitors^5,10^. Most of the serpins share identical structures with seven or nine α-helices and three β-sheets. Serpin features the property of a reactive center loop (RCL) that binds to objective protein near the C terminus of the sequence^11^. Serpin RCL possesses a scissile bond between residues P1 (N-terminus of cleavage event) and P1′ (C-terminus of cleavage event). Once scissile bond is cleaved, a dramatic conformational change is triggered. Serpin together with its peptide ultimately forms a permanent covalent complex. In comparison to serpins, the canonical protein inhibitors usually occur as single, small proteins and are composed of several disulfide bonds. Through an exposed RCL, serpin can interact with the protease reactive center region and defunctionalize the activity of the proteases. The mechanism of canonical protein inhibitors is similar to that in serpins, since both of them can bind to the objective proteins in protease-substrate interaction mode. The difference between them is that canonical protein inhibitors are kept in reversible tight-binding interactions. Canonical inhibitors can be divided into about 20 protein families such as Kazal, trypsin inhibitor-like (TIL), Pacifastin and Kunitz serine protease inhibitors^5^. The A2Ms are much larger proteins discovered in living organisms and vary widely from invertebrates to vertebrates. The A2Ms are members of thioester-containing proteins (TEPs)^12^. There are six members in TEPs family, including C3/C4/C5, A2M, pregnancy zone protein (PZP), CD109, insect TEP (iTEP), and the complement 3 and PZP-like A2M domain-containing 8 (CPAMD8)^13,14^. Other than the inhibitors mentioned above, A2M could trap the target proteases by a α_2_-macroglobulin cage to form a reversible complex. As a result, proteases from different classes such as thiol-, carboxyl-, serine- and metalloprotease can be enfolded by A2Ms^15^.

Genetic analyses have provided fundamental understanding of functions of these SPIs in immunity, embryo development and reproduction. It is widely studied that serpins take effect in inhibiting Spätzle-processing enzyme in Toll signaling pathway and PPO activation cascade^16^. In *Manduca sexta*, serpin-4 (MsSRPN4) and serpin-5 (MsSRPN5) are inhibitors of hemolymph proteinase-1(MsHP-1) and hemolymph proteinase-6 (MsHP-6), and function in protease cascades of PPO pathway. Thus, MsSRPN4 and MsSRPN5 may be involved in melanization processes^17^. *Manduca sexta* serpin-1J (MsSRPN1J) and serpin-6 (MsSRPN6), capable of forming covalent complexes with prophenoloxidase-activating proteinase-3 (MsPAP3) and hemolymph proteinase-8 (MsHP-8), may participate in the Toll pathway and PPO activating cascades^18,19^. Studies also showed that serpins mediate several anti-inflammatory effects in host-pathogen interactions^20^. In addition, some serpins even inhibit cysteine proteinase through an alternative mechanism^21^. Compared to serpins, there are fewer molecular studies on canonical SPIs. A Kazal-type serine protease inhibitor named oryctin inhibits α-chymotrypsin, endopeptidase K, subtilisin and elastase, indicating it may be related to digestion^22^. Pacifastin-like peptides can induce significant growth retardation in a desert locust^23^ and can defend against entomopathogenic fungi in the silkworm^24^. In some mosquitos and ticks, canonical SPIs secreted by salivary or venom gland possess anticoagulation activity, which is vital for these blood-feeding insects to successfully manipulate the physiology of hosts^8,25^.

Recently, genome-wide analyses have helped us to identify serpin genes from several insect species, including *Anopheles gambiae* and *Aedes aegypti* ^26^, 12 *Drosophila* species^27^, *Apis mellifera* ^28,29^, *Tribolium castaneum* ^30^, *Bombyx mori* ^31^, and *Plutella xylostella* ^32^. Compared to serpins, the comprehensive analyses of all the SPI genes based on genome databases have only been illustrated in two lepidopteran species (*B. mori* and *P. xylostella*) so far^26,33^. Since all of the SPIs play significant roles in several biological processes, identification of these regulator molecules is necessary for better understanding of the biochemical and molecular functions of SPIs.

In this study, *Pteromalus puparum* SPI genes were identified and characterized based on newly sequenced *P. puparum* genome. *P. puparum* is a pupal endoparasitoid wasp and plays a critical role in biological control of certain pierid species, especially the small white butterfly, *Pieris rapae*. Since *P. rapae* is an important pest of the crucifer and caper families, studying on this parasitic wasp is of great significance. Besides, we have finished the whole *P. puparum* genome and several transcriptomes (unpublished data). Serine proteases and their homologs, serving as the putative targets of SPIs, have already been characterized in *P. puparum* genome^34^. Thus, this information provided us a solid foundation to explore SPI genes of *P. puparum*. In the present study, *P. puparum* SPIs and their putative functions were identified and predicted. Meanwhile, the temporospatial expression analyses of SPI genes were also performed. These findings give us a detailed overview of the *P. puparum* SPIs for further clarifying their functions in digestion, innate immunity, development and reproduction.

## Results

### Identification of SPI genes in *P. puparum*

We obtained 57 SPI genes in total through BLAST searches in *P. puparum* genome database (Table 1 & Supplementary Table S1). All of the SPI amino sequences were summarized in Supplementary Table S2. The SPI genes of an ectoparasitoid wasp named *Nasonia vitripennis* were also listed for comparative purpose (Supplementary Table S3). These 57 *P. puparum* SPIs (PpSPIs) contain 7 inhibitor domains and can be divided into three types: serpin, A2M and canonical SPIs (Table 1).

**Table 1:** Serine protease inhibitor (SPI) domains in *Pteromalus puparum*.

Gene Name	SPI domain	Number of Domain	Category
**PpSPI1~PpSPI2**	serpin	1	serpin
**PpSPI3**		2	
**PpSPI4~PpSPI10**		1	
**PpSPI11~PpSPI12**	Kazal	1	Canonical SPIs
**PpSPI13**		3	
**PpSPI14~PpSPI26**		1	
**PpSPI27**		11	
**PpSPI28**		3	
**PpSPI29~PpSPI30**		1	
**PpSPI31**		8	
**PpSPI32**		1	
**PpSPI33~PpSPI34**	Pacifastin	1	
**PpSPI35**		7	
**PpSPI36**		8	
**PpSPI37**		5	
**PpSPI38**		9	
**PpSPI39**		4	
**PpSPI40**		2	
**PpSPI41~PpSPI42**		1	
**PpSPI43**		2	
**PpSPI44**		1	
**PpSPI45**		2	
**PpSPI46~PpSPI50**	TIL	5	
**PpSPI51~PpSPI53**	Kunitz_BPTI	3	
**PpSPI54**	Kunitz_BPTI/WAP	11/1	
**PpSPI55~PpSPI57**	A2M	1	A2M

### Serpins

A total of 10 serpin genes of *P. puparum* were characterized in this study. BLAST searches for serpin genes were also conducted in *N. vitripennis* genome, which revealed a similar number of serpins (9) (Supplementary Table S3). The length of *P. puparum* serpins varies from 150 to 830 amino residues, with a typical mature serpin about 300–400 amino acids. PpSPI10 is incomplete in part of the amino acid sequence whereas PpSPI3 contains two intact serpin domains, resulting in abnormal sequence length (Supplementary Table S1). The structures of serpins determine their mechanisms in protease inhibition. The most important regions involved in the conformational changes of serpin in proteinase inhibition are beta-sheet A and RCL. We aligned *P. puparum* serpins with structurally well-defined inhibitory serpins (*Manduca sexta* serpin 1 K and human alpha-1 antitrypsin)^35,36^, and marked the structures, beta-sheets, and the hinge, breach, shutter and gate regions accordingly (Supplementary Figure S1). Analysis of the serpin molecular structure showed that three alpha-helixes and nine beta-sheets are conserved in most of *P. puparum* serpins. The sequence of PpSPI6 is distinguished from other *P. puparum* serpins by missing the definitive amino acids in hinge, breach, shutter and gate regions. PpSPI4 contains nearly 80 residues between helix I and strand 5 A while PpSPI10 loses the structure from helix C to strand 3 C.

**Figure 1:**
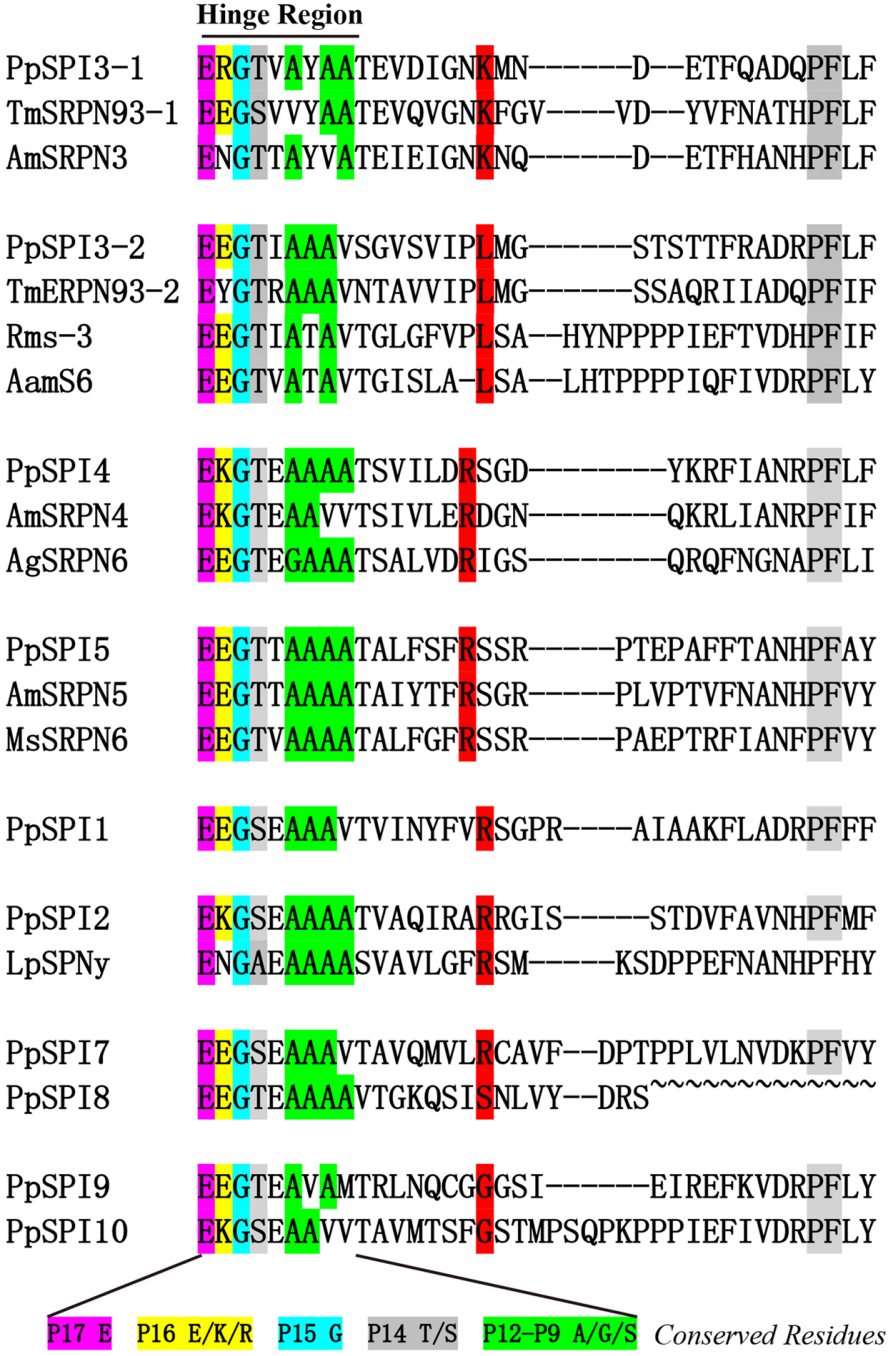
Multiple sequence alignment of the hinge and reactive center loop (RCL) regions of *Pteromalus puparum* serpins. The hinge and RCL regions of *P. puparum* serpins were aligned with those serpins with known functions. Predicted P1 residues are highlighted in red. *P. puparum* serpins are presented in numerical order and grouped with homologous serpins from other insects determined by phylogenetic analysis. The GenBank accession number for sequences used in phylogenetic analysis in Fig. 1: AamS6 (ABS87358.1, *Amblyomma americanum*); AmSRPN3, AmSRPN4, AmSRPN5 (XP_001122067.2, XP_003249882.1, XP_006562425.1, *Apis mellifera*); AgSRPN6 (ABJ52806.1, *Anopheles gambiae);* LpSPNy (ACQ83466.1, *Leptopilina boulardi*); MsSRPN6 (AAV91026.1, *Manduca sexta*); Rms-3 (AHC98654.1, *Rhipicephalus microplus*) and TmSPN93 (BAL03254.1, *Tenebrio molitor*).

The hinge region, which stabilizes the metastable native serpin conformation, is located at N-terminal portion of the RCL region. The consensus patterns of specific amino acids in the hinge region were observed in most of the *P. puparum* serpins except PpSPI6. The amino acid at P1 position determines the enzyme inhibition specificity of the serpin. Serpin with Arg or Lys at the P1 position may prohibit trypsin-like SPs, may have the inhibitory activity to chymotrypsin-like SPs with Phe, Tyr, Leu or Ile at P1 position, and may participate in inhibiting elastase-like enzyme with P1 position of Ala or Val^26,33^. We predicted the position of the scissile bond of the *P. puparum* serpins (Fig. 1). PpSPI1, 2, 3-1, 4, 5 and 7 are anticipated to inhibit trypsin-like SPs with P1 position containing Arg or Lys. PpSPI3-2 has a Leu residue at P1 position, and may serve as a chymotrypsin inhibitor.

**Figure 2:**
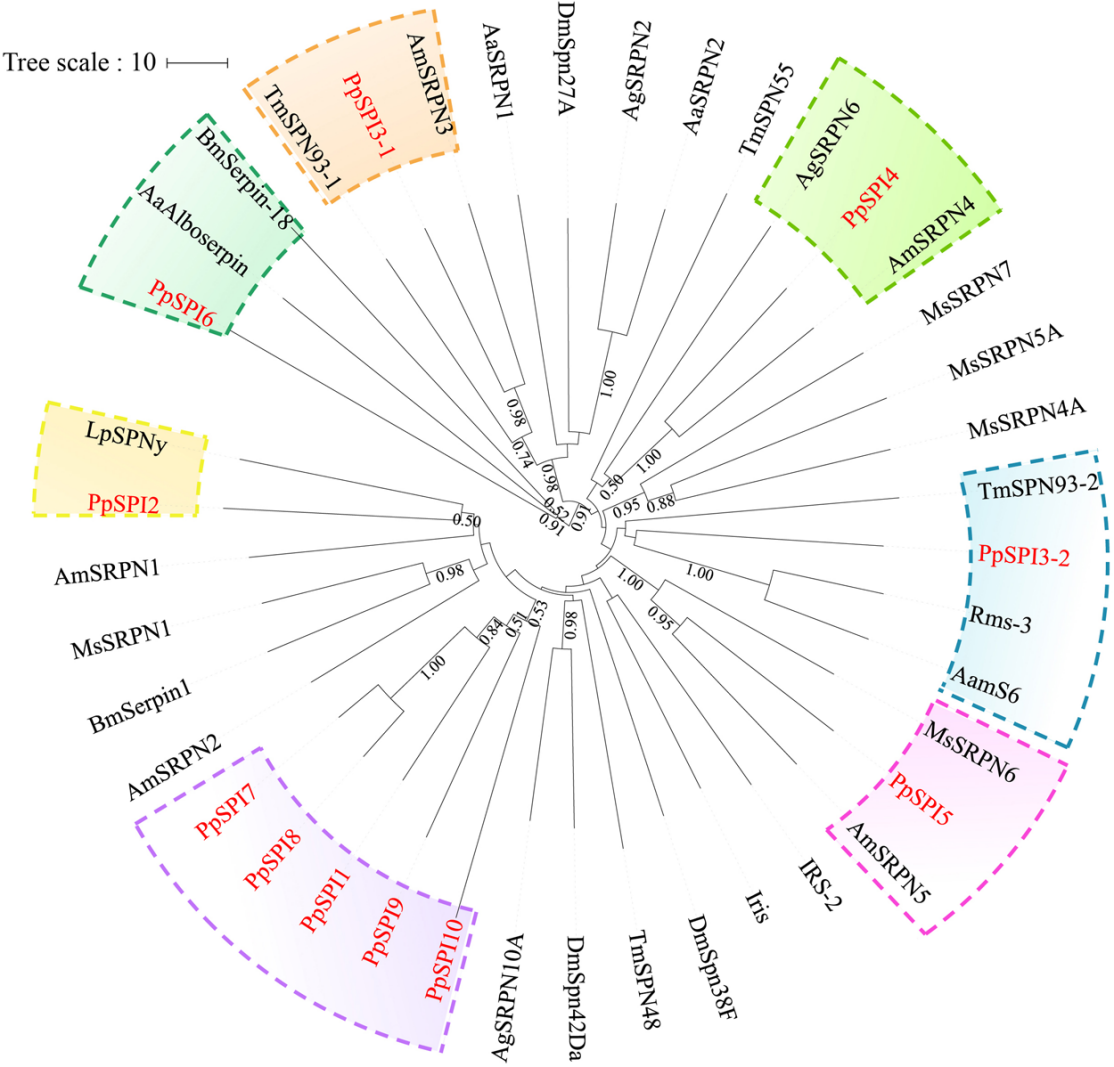
The phylogenetic tree includes 11 *Pteromalus puparum* serpins, 5 *Apis mellifera* serpins and 24 known function serpins from 10 other insect species. All of the serpins were named according to relevant literatures. Phylogenetic tree was constructed by Neighbor-joining method, using the program Mega 5.10. The bootstrap values greater than 0.5 are dotted on the nodes. The GenBank accession numbers for sequences used in phylogenetic analysis in Fig. 2: AaSRPN1, AaSRPN2, AaAlboserpin (XP_001648011.1, XP_001651231.2, AAC31158.1, *Aedes aegypti*); AamS6 (ABS87358.1, *Amblyomma americanum*); AgSRPN2, AgSRPN6, AgSRPN10A (ABJ52801.1, ABJ52806.1, XP_314159.2, *Anopheles gambiae*); AmSRPN1, AmSRPN2, AmSRPN3, AmSRPN4, AmSRPN5 (GB17012^33^, XP_016772722.1, XP_001122067.2, XP_003249882.1, XP_006562425.1, *Apis mellifera*); BmSerpin1, BmSerpin-18 (ACT36272.1, ACG61181.1, *Bombyx mori*); DmSpn27A, DmSpn38F, DmSpn42Da (NP_652024.1, CAB63098.1, NP_724512.1, *Drosophila melanogaster*); Iris, IRS-2 (CAB55818.2, ABI94056.2, *Ixodes ricinus*); LpSPNy (ACQ83466.1, *Leptopilina boulardi*); MsSRPN1, MsSRPN4A, MsSRPN5A, MsSRPN6, MsSRPN7 (AAC47341.1, AAS68503.1, AAS68507.1, AAV91026.1, ADM86478.1, *Manduca sexta*); Rms-3 (AHC98654.1, *Rhipicephalus microplus*); and TmSPN48, TmSPN55, TmSPN93 (BAI59108.1, BAI59107.1, BAL03254.1, *Tenebrio molitor*).

Phylogenetic tree was constructed using *P. puparum* serpins along with 5 *A. mellifera* serpins and 24 known function serpins from 10 insect species (Fig. 2). The serpins from *P. puparum* form 7 clades. PpSPI3 is a twin-domain serpin, with the first domain PpSPI3-1 clusteres with AmSRPN3 and TmSPN93-1, the second PpSPI3-2 forms clades with AamS6, Rms-3 and TmSPN93-2. PpSPI3-1 and PpSPI3-2 shared the same amino acids at P1 position (Lys) with the corresponding genes, respectively. PpSPI4 shares a high similarity with AmSRPN4 and AgSRPN6. Sequences alignment showed that the hinge region and the residue at P1 position (Arg) of AgSRPN6 are identical to that of PpSPI4 (Fig. 1). We examined the microbe-induced expressions of *PpSPI4* using Gram-negative bacterium *Escherichia coli*, Gram-positive bacterium *Micrococcus luteus* and entomopathogenic fungus *Beauveria bassiana* (Supplementary Figure S2). The expression of *PpSPI4* could be induced by the treatment of *B. bassiana* 24 h post infection (p. i.). PpSPI5, AmSRPN5 and MsSRPN6 form a clade with supported bootstrap values no less than 95%. PpSPI5 and MsSRPN6 share the same amino acids at P1 position (Arg) and hinge position (Fig. 1). Quantitative real-time PCR (qPCR) analyses indicated a dramatically increased expression of *PpSPI5* in all of three microbes-infected cases. The induction was stable after *E. coli* infection whereas the expression of *PpSPI5* was highly induced in the *M. luteus*-infected and *B. bassiana*-infected samples 6 h and 24 h p. i., respectively (Supplementary Figure S2).

### Canonical serine protease inhibitors

**Figure 3:**
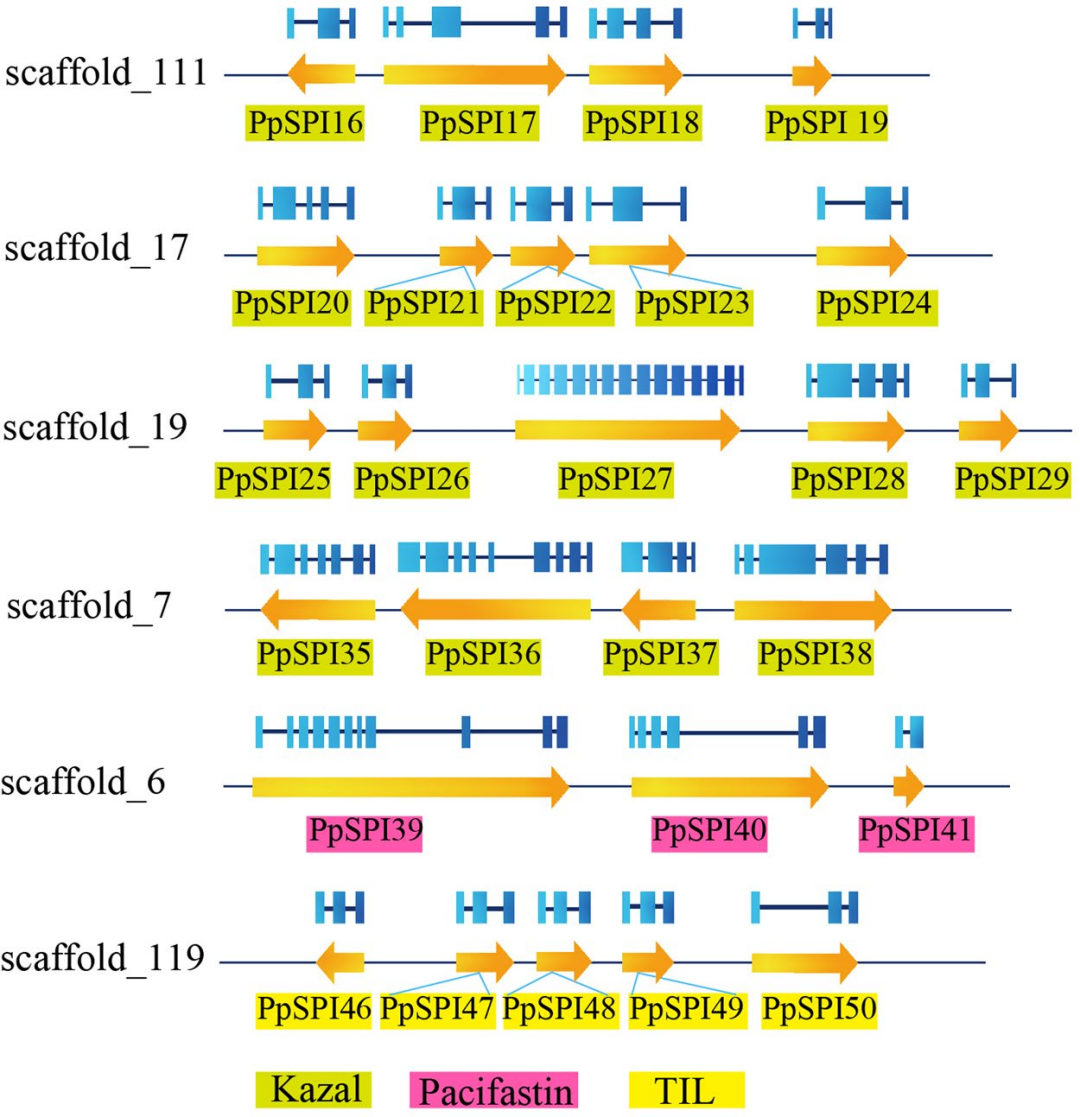
Structure and location of *Pteromalus puparum* canonical SPI genes on scaffolds. The arrows indicate the transcription orientations and gene sizes on scaffolds. The exons are shown with blue boxes.

There were 44 canonical SPI genes identified in *P. puparum*. The canonical SPIs in *P. puparum* can be divided into 5 different families: Kazal, TIL, Pacifastin, Kunitz and whey acidic protein (WAP) (Table 1). Most of these canonical SPIs only contain one kind of SPI domains except PpSPI54. Canonical SPIs are cysteine-rich peptides and common with multiple disulfide bonds buried inside the molecule to offer structure stability^5^. Multiple sequence alignments in the same SPI family showed conservation in the number and arrangement of the cysteine residues (Supplementary Figure S3). Kazal, Pacifastin and Kunitz domain contain 3 pairs of disulfide bonds (with the combination form of 1–5, 2–4 and 3–6 in Kazal, 1–6, 2–4 and 3–5 in Kunitz, 1–4, 2–6 and 3–5 in Pacifastin), 4 pairs in WAP (linking 1–6, 2–7, 3–5, 4–8) and 5 pairs in TIL (ranging from 1–7, 2–6, 3–5, 4–10 and 8–9). SPIs from the same family show gene cluster phenomenon in scaffolds. There are 4, 5 and 5 Kazal genes clustered in scaffold_111, _17 and _19, respectively. The same things also happen in Pacifastins family. Three members in Pacifastin family are located in scaffold_6 and another 4 members located in scaffold_7. Studies also showed all of the 5 TIL genes are clustered in scaffold_119, sharing similar molecular sizes and intron-exon structures (Fig. 3).

Canonical SPI interacts with its substrate via a convex, exposed binding loop. The loop has an active P1-P1′ scissile bond in the cleavage site of the SPI. P1 residue determines the specific inhibitory activities of the SPIs, which are similar to the roles of P1 in serpins^6^. We listed the putative P1 residue through the alignments of SPI domains (Supplementary Figure S3). Of 43 canonical SPIs, 28 are single domain proteins and their inhibition activities depend on the characteristics of single SPI domain. According to sequence alignments, PpSPI35, PpSPI46 and PpSPI57 may inhibit trypsin-like SPs (with Arg located at P1 position); PpSPI31 is anticipated to inhibit elastase-like SPs (with Ala located at P1 position); PpSPI28 and PpSPI48 may participate in inhibiting chymotrypsin enzymes (with Phe located at P1 position).

**Figure 4:**
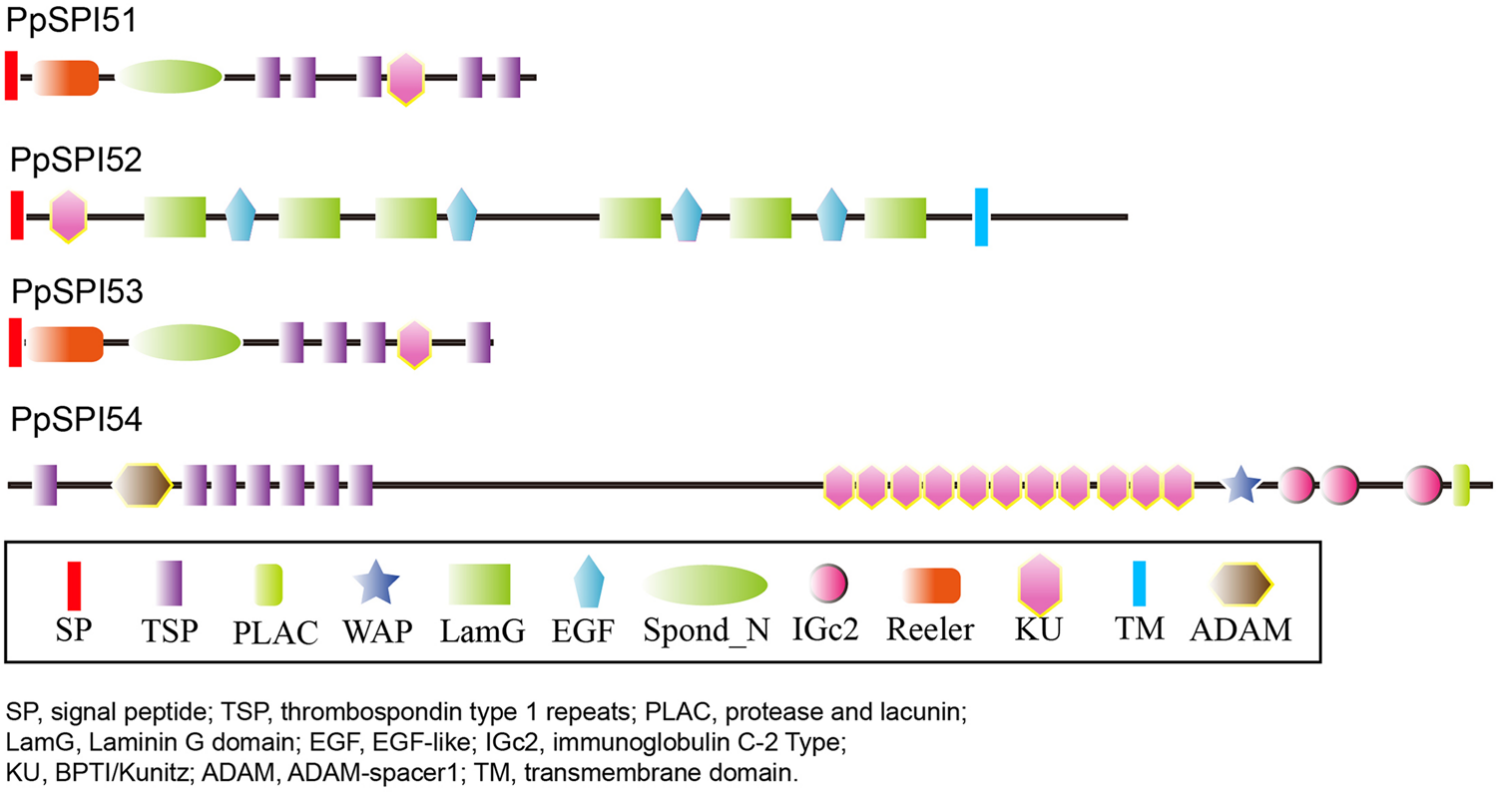
Domain organizations of *Pteromalus puparum* SPIs with non-inhibitory units in the Kunitz family.

Fifteen canonical SPIs encompass multi domains. SPIs in Kazal, Kunitz and Pacifastin families contain several repeats of homogeneous SPI domains. SPIs with multiple SPI domains come along with varieties of P1 positions, so these SPIs exhibit inhibitory activities. PpSPI29 may inhibit all of the trypsin, chymotrypsin and elastase enzymes, due to Ile and Phe at the second and tenth P1 position, Ala and Val at the third and sixth P1 position, and Arg and Lys at the fourth and seventh P1 position. PpSPI37, 38, 39, 40, 41 could also inhibit both chymotrypsin and trypsin according to the corresponding amino acids at P1 positions (Supplementary Figure S3). We also analyzed the structure domains of *P. puparum* Pacifastins and predicted putative dibasic cleavage sites based on the liable fracture site (RR, RK or KK residues) (Supplementary Figure S4). Several smaller, single or multi domain inhibitors were obtained, and each can exert function alone. The SPIs in Kunitz and WAP family have the mixtures of SPI domains and non-inhibitor domains (Fig. 4). PpSPI51 and PpSPI53 show similarity in structural arrangement. PpSPI53 consists of 1 Reeler, 1 Spond_N, 4 TSP and 1 Kunitz_BPTI domains, whereas PpSPI51 has one added TSP domain in C-terminal amino sequences. PpSPI52 includes 1 Kunitz_BPTI, 4 EGF and 6 Laminin G domains. PpSPI54 is composed of 7 TSP, 11 Kunitz_BPTI, 3 immunoglobulin C-2, 1 ADAM-spacer1, 1 WAP and 1 PLAC domains.

### A2M

**Figure 5:**
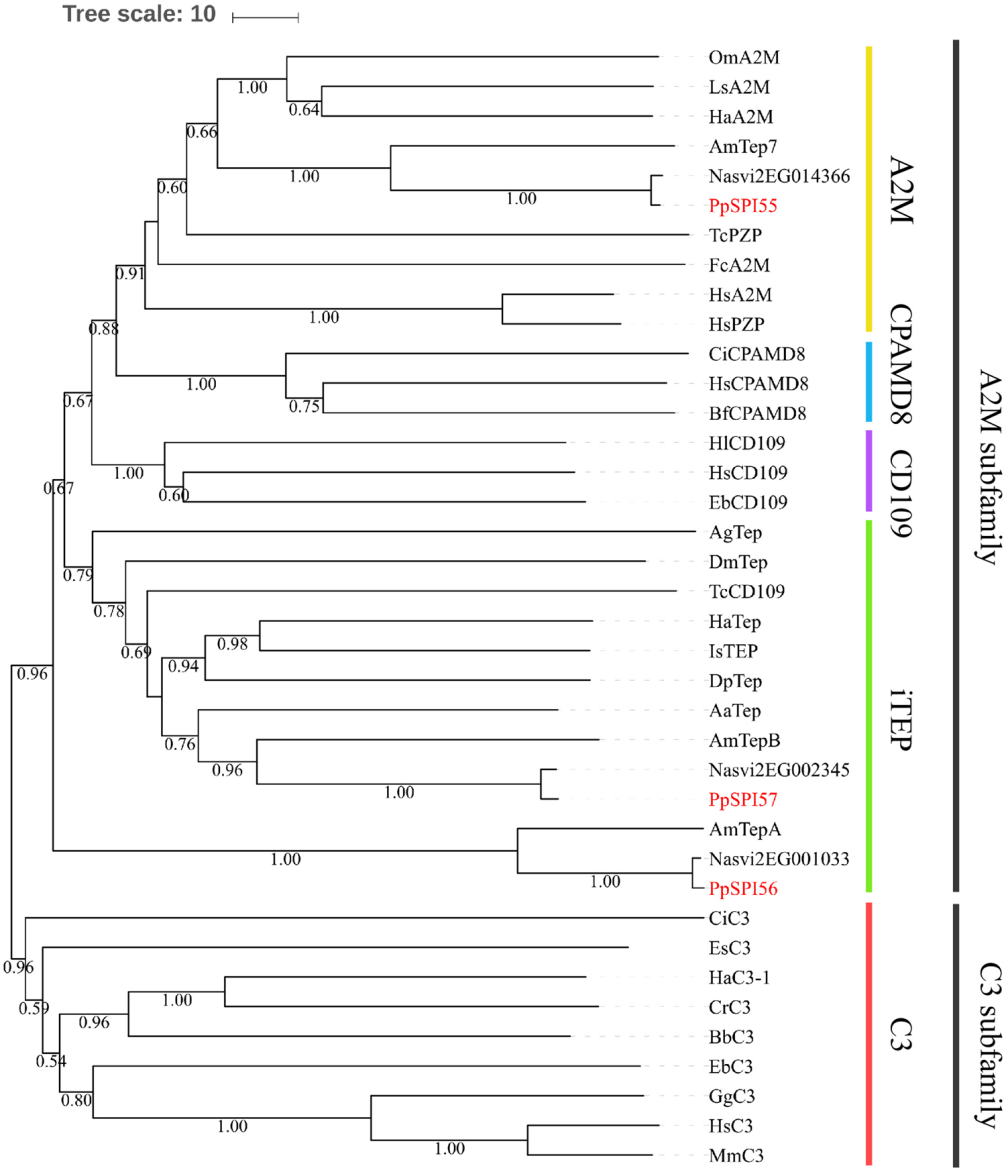
Phylogenetic analysis of thioester-containing proteins. Phylogenetic tree was constructed by Neighbor-joining method, using the program Mega 5.10. The bootstrap values greater than 0.5 l are presented on the nodes. The first two letters in each of the serpins represent the acronym of scientific name for a given species. The GenBank accession number for sequences used in phylogenetic analysis in Fig. 5: AaTep (EAT39604.1, *Aedes aegypti*); AgTep (AAG00600.1, *Anopheles gambiae*); AmTepA, AmTepB, AmTep7 (XP_397416.3, GB12605^28^, XP_006565503.1, *Apis melliferal*); BbC3 (AB050668.1, *Branchiostoma belcheri*); BfCPAMD8 (XP_002586872.1, *Branchiostoma floridae*), CiCPAMD8, CiC3 (XP_009861615.2, NP_001027684.1, *Ciona intestinalis*); CrC3 (AAQ08323.1, *Carcinoscorpius rotundicauda*); DmTep (NP_523578.1, *Drosophila melanogaster*); DpTep (EFX86067.1, Daphnia pulex); EbCD109, EbC3 (BAD12264.1, P98094.1, *Eptatretus burgeri*); EsC3 (ACF04700.1, *Euprymna scolopes*); FcA2M (ABP97431.1, *Fenneropenaeus chinensis*); GgC3 (U16848.1, *Gallus gallus*); HaA2M, HaC3–1, HaTep (AB622470.1, AB622468.1, AB622471.1, *Hasarius adansoni*); HlCD109 (AB481386.1, *Haliplanella lineata*); HsA2M, HsCD109, HsCPAMD8, HsC3, HsPZP (P01023.3, NP_598000.2, NP_056507.2, NP_000055.2, CAA38255.1, *Homo sapiens*); IsTEP (XP_002409560.1, *Ixodes scapularis*); LsA2M (BAA19844.1, *Limulus sp*.); MmC3 (NM_009778.3, *Mus musculus*); OmA2M (AAN10129.1, *Ornithodoros moubata*) and TcPZP, TcCD109 (XP_008195453.1, XP_972838.2, *Tribolium castaneum*).

The A2Ms belong to the TEP family. TEPs usually contain A2M-N, A2M-N2, A2M, A2M-COMP and A2M-RECEP domains. In the genome of *P. puparum*, we identified three SPIs containing typical featured domains of TEPs. Phylogenetic tree was constructed using three *P. puaprum* SPIs and several TEP sequences from 22 different species by NJ method (Fig. 5). TEPs are separated into C3 and A2M subfamilies, both of which are supported by 96% bootstrap values. The subfamily of A2M is then categorized into four parts, A2M, CPAMD8, CD109 and iTEP. SPIs from *P. puparum* share high similarity with corresponding protein sequences from *N. vitripennis* and *A. mellifera* with 1:1:1 ratio of ortholog. In detail, PpSPI55 clusters with A2Ms whereas PpSPI57 clusters with insect TEPs. PpSPI56, together with corresponding SPIs in *N. vitripennis* and *A. mellifera*, forms a branch in A2M subfamily, distinct from other A2M subfamily genes in vertebrate and invertebrate. Based on the overall topology of the tree, PpSPI56 is most likely to be an iTEP. Therefore, we considered PpSPI55 as an A2M, PpSPI56 and PpSPI57 as iTEPs.

To classify the conserved and diverged residues of three *P. puparum* A2M and iTEP genes, multiple sequence alignments were performed. PpSPI55, PpSPI56 and PpSPI57 possess putative signal peptides in the N-terminus for secretion. In addition, four proteinase-binding A2M domains (A2M-N, A2M-N2, A2M and A2M-COMP) and one receptor binding A2M domain (A2M-RECEP) were characterized (Supplementary Figure S5). The sequences between *P. puparum* A2M and iTEP with corresponding *N. vitripennis* genes share significant similarity with the identities no less than 97%. The FPETW conserved in bait region of A2M family, also exists in the sequences of PpSPI55. The thioester motifs in PpSPI55 and PpSPI57 are GCGEQ, which are also identical to those of other TEPs. The corresponding position in the sequences of PpSPI56 is replaced by DCGEQ. His (H) residue locates at approximately 100 amino acids downstream of the thioester motif, may influence the specific binding activity of the A2M^37^. However, Asp occupies this conserved site in PpSPI56. We also observed an excessive cysteine-rich C-terminal extension in PpSPI55 and corresponding gene in *N. vitripennis*.

### Developmental and sex-specific expression

**Figure 6:**
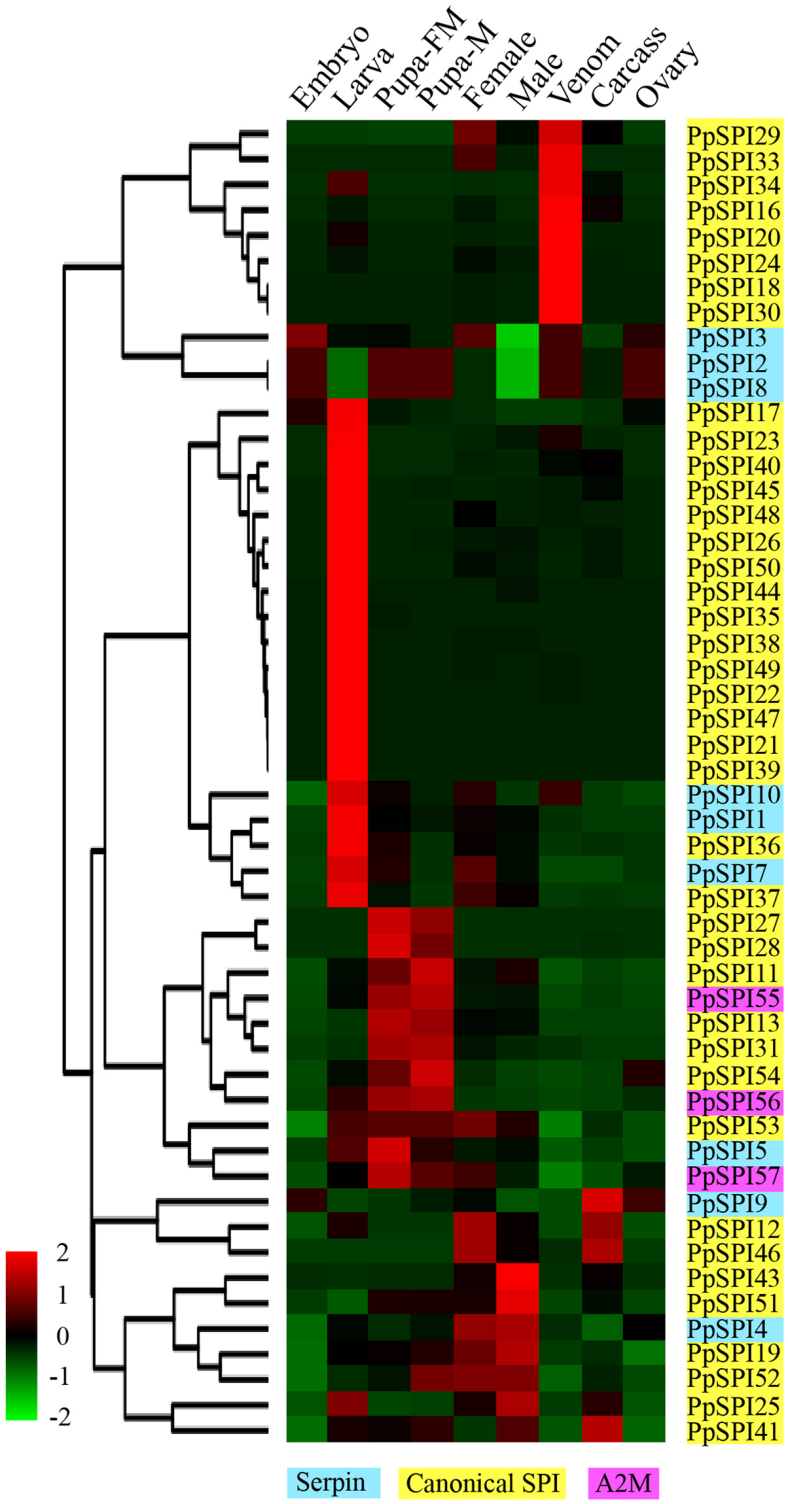
Expression profiles of *Pteromalus puparum* SPI genes across different developmental stages and tissues. Log_2_ FPKM values for the SPIs are presented by bar colors where the darker red represent higher expression values, the darker green represent lower expression values.

We analyzed the expression patterns of the *P. puparum* SPI genes in different developmental stages (embryo, larva, pupa and adult) and different tissues (ovary, venom gland and carcass without venom gland). The transcriptome data enable us to calculate FPKM values of the entire SPI genes (Supplementary Table S4). *PpSPI6*, *PpSPI14*, *PpSPI15*, *PpSPI32* and *PpSPI42* with very low FPKM values in all these existing databases may be pseudogenes. We retained these SPI genes in consideration of that some genes were only highly expressed in a specific tissue, but not or barely expressed in other tissues. The expression levels of 52 *PpSPI* genes (with FPKM values greater than 1) were profiled by all of nine transcriptomes (Fig. 6).

**Figure 7:**
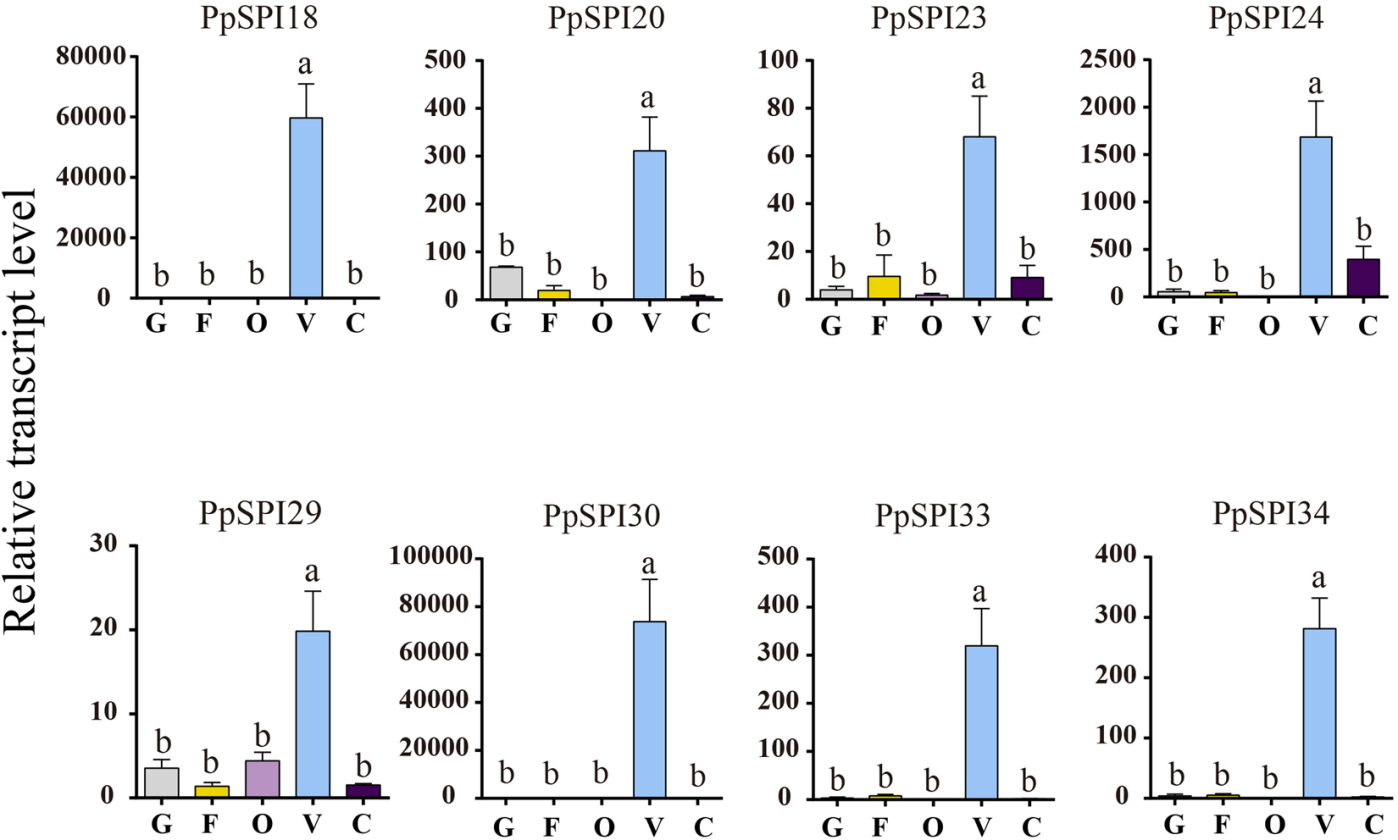
Tissue-specific expressions of *Pteromalus puparum* SPI genes. Total RNA was extracted from the gut (G), fat body (FB), ovary (O), venom (V) and carcass (C, the remaining body) of the adult female *P. puparum*, and used to analyze the expression patterns of these SPIs using qPCR. *P. puparum* 18 s rRNA was used as a housekeeping gene. Error bars represent the means ± standard deviations from three biological replicates. A one-way ANOVA was used to determine the significant difference with different lowercase letter (a–c) (*p* < 0.05).

The results showed that *P. puparum* SPI genes exhibited stage-, sex- and tissue-specific expression patterns. The PpSPI gene expression patterns can be roughly divided into two groups. In the first group, 6 Kazals and 2 Pacifastins are most abundant in venom gland. The venom serves a decisive role in parasitism, and the expressions of SPI genes in venom gland are our major focus. We also used the same methods mentioned before^38^ to define specifically expressed genes in the venom gland and the analysis yielded 8 venom-specific SPI genes, with *PpSPI16* replaced by *PpSPI23* (Supplementary Table S5). qPCR results of these venom gland specifically expressed genes are consistent with the transcriptome data (Fig. 7). Furthermore, four of eight SPIs also exist in the venom gland proteome and were considered as venom proteins (Supplementary Table S5). SPI genes in the second group form three clusters that were highly expressed in larval, pupae and adult stages, respectively. The SPI genes in the first cluster presented distinct expressions in larva stage, of which canonical SPI genes occupy 17 out of total 20 genes. Eleven SPI genes including all of three A2M-like genes showed relative high expressions in female and male pupae and belong to the second cluster. The last presented genes were mainly expressed in carcass, female adult and male adult. The expression profiles of SPI genes in different development stages were also verified by qPCR results, and the expression patterns were parallel to the transcriptome data (Supplementary Figure S6).

## Discussion

In the genome of *P. puparum*, total 57 SPI genes belonging to 7 families (serpin, Kazal, Pacifastin, TIL, Kunitz, WAP and A2M) were identified. The comprehensive analyses of SPIs have been conducted in two lepidopteran insects, with 80 SPI genes in *B. mori* and 61 in *P. xylostella* ^26,33^. The number of *P. puparum* SPI genes is less than that in *B. mori* or *P. xylostella*. The differences in the numbers of canonical SPI families are also showed between *P. puparum* and lepidoptera insects. Four families of canonical SPI genes (WAP, WAP/Antistasin/Kunitz, amfpi and Bowman-Birk) identified in the lepidoteran insects are absent in *P. puparum* genome. SPI genes in *N. vitripennis* are precisely similar to *P. puparum* SPI genes in the quantity and the ratio (Supplementary Table S3). The analysis of scaffold location revealed that tandem repeats of genes with similar structure are observed in *P. puparum* TIL, Pacifastin and Kazal genes. This is also a common event identified in several species, not just restricted to SPI gene families^39^. The expansion of SPI sequences in one scaffold may be the result of gene duplication during quick evolution.

Besides the gene duplication, diversity of the SPIs can also be generated post transcriptionally or post translationally. For some insect serpins, alternate splicing of an exon that encoded the RCL regions gives rise to several serpins with different inhibitory activities. *MsSRPN1* is a good example for this mechanism, which contains 14 copies of its 9th exons for different carboxyl-terminal residues^40,41^. The serpin-1 from *B. mori* orthologous to *MsSRPN1*, contains four alternative splices of the exon 9^27,42^. Similar situation can also be identified in the hymenoptera wasps, *PpSPI1* ^43^ with 16 putative splicing isoforms sharing the first seven exons, but differing in the RCL encoded exon 8, resulting in diverse inhibitors. Previous studies have reported that posttranslational modification is common in Pacifastins family^24^. Members in Pacifastin families generally contain one signal peptide followed by several Pacifastin-like domains^44^. These Pacifastins could be divided into several smaller inhibitor peptides by putative dibasic cleavage sites. The cleavages of multi-domain Pacifastin inhibitors and the structural organizations are similar to the mechanisms in typical endocrine or neuronal peptide precursors, which could be broken into small peptides by endoproteinases through pairs of basic amino acids^45,46^. Further investigation should be used to detect the putative enzyme and the possible physiological role of the cleavage of these multiply-domain Pacifastins. Analyses of Pacifastin sequences in *P. puparum* showed that they can transport extracellularly and be divided into several smaller peptides by dibasic cleavage sites.

Serpins are essential components in immune response, with their role being regulatory control over Toll pathway^18,47^ and PPO activation by inhibiting serine protease cascades^16^. Limited studies have also shown that serpins play a role in development and reproduction^48,49^. A total of 10 serpins were identified in the genome of *P. puparum*. Most of these serpins contain conserved beta-sheet and alpha-helix structures. PpSPI6 distinguished with other *P. puparum* serpins in sequences. The phylogenetic tree showed PpSPI6 forms a clade distant from other serpins. These results indicated PpSPI6 may not be a typical inhibitor. Genetic and biological studies could be used to test the biochemical roles of PpSPI6 in further research. The rest seven of nine *P. puparum* serpins may function as serine protease inhibitors, conformed to the sequence alignments and putative dibasic cleavage sites. PpSPI3 consists of two serpin domains, sharing similarity in hinge regions and cleaved sites with a twin-domain serpin TmSPN93. It was reported that TmSPN93 isolated from a large beetle *Tenebrio molitor*, also contains two complete, tandemly arrayed serpin domains. The first serpin domain of TmSPN93 could bind to Spätzle-processing enzyme to form a permanent protein complex whereas the second serpin domain forms a stable complex with Spätzle activating enzyme, both of which were involved in the Toll signaling cascade^47^. Although *P. puparum* and *T. molitor* have distant correlation between relatives, they display the same dual-domain organizations, and similar amino acids in hinge regions and P1 position (Fig. 1). Therefore, we inferred PpSPI3 may modulate relative serine proteinases in the Toll activation pathway as TmSPN93. PpSPI4 shares a high similarity with AmSRPN4 and AgSRPN6. Previous studies indicated that AgSRPN6 could inhibit bovine pancreatic trypsin *in vitro* ^50^. Since AgSRPN6 and PpSPI4 share the same residue at P1 position (Arg), PpSPI4 may serves as a trypsin-inhibitor. The experiment also showed the expression profile of *AgSRPN6* was significantly induced after infection. Knockdown of the expression level of AgSRPN6 by RNA interference delayed progression of parasite lysis as well as increases number of melanized parasites^50,51^. We examined the microbe-induced expressions of *PpSPI4*. qPCR showed that *PpSPI4* can be highly induced p. i. PpSPI5 clusters with MsSRPN6. In *M. sexta*, MsSRPN6 can covalently link with MsPAP3 or MsHP-8. MALDI-TOF-MS showed that the cleavage site of MsSRPN6 is between Arg and Ser^19^. PpSPI5 and MsSRPN6 share the identical amino acids (Arg and Ser) at P1 position of the cleavage site and hinge region (Fig. 1), which further indicated the result that PpSPI5 may inactivate its cognate enzyme in the scissile bond between residues Arg and Ser. An induction pattern of *MsSRPN6* was observed after bacterial or fungus infection. We also detected the expression profiles of *PpSPI5*. The expression levels of *PpSPI5* increased strikingly after immune challenges. Besides, previous studies have demonstrated that AgSRPN6 and MsSRPN6 participate in regulating the PPO activation pathway^19,51^. Based on sequence alignment, phylogenetic tree and expression patterns, we inferred that PpSPI4 and PpSPI5 may also participate in the PPO activation pathway.

Apart from serpins, canonical SPIs with single or multiply domains were characterized. Among them, three SPIs are considered as mixed type inhibitors for containing non-inhibitor domains. PpSPI52 is a protein with unknown function. PpSPI54 is similar to papilin, an extracellular matrix glycoprotein involved in cell rearrangements of different matrices in embryo development and may modulate metalloproteases^52^. PpSPI51 and PpSPI53 are homologs to F-spondin. Previous studies revealed F-spondin serves as an extracellular matrix that attached molecule to promote neuritis commissural and inhibit the motor axons^53^.

There were three SPI genes containing the characteristic of A2M domains identified in *P. puparum* genome. We made sequences alignment and phylogenetic tree, which would be useful in better understanding of this family. The thioster domain GCGEQ and His residue 100 amino acids downstream of the thioester are conserved residues in most of the TEPs. However, DCGEQ and Asn replace these two conserved sites in PpSPI56. Besides, phylogenetic tree showed PpSPI56 was divided into a branch far away from other A2Ms. The differences in the potentially important sites and topology of the tree indicated the function of PpSPI56 divergent from other A2Ms^54^. PpSPI55 possesses of both of the conserved motifs. An excessive cysteine-rich C-terminal extension was also observed in PpSPI55. The structure difference in C-terminal of the A2M sequences is also popular in ant, wasp and honey bee A2M. However, it is not observed in other insect A2Ms^55^. Based on the typical thioester motif and the catalytic His residue, PpSPI55 may show a characteristic biological function as a typical A2M, but the function of unusual C-terminal extensions is difficult to predict.

RNA-seq databases enable us to detect gene temporospatial expression patterns. Gene expression profiles showed that 22 SPI genes were highly expressed in the larval stage, occupying for nearly 40% of the whole SPI genes. The expression patterns were extremely parallel to 53 out of 133 serine protease genes previously found in *P. puaprum* ^34^. The wasps secreted massive proteinases in food digestion for its rapid growth. The sufficient SPIs are necessary in immobilizing excessive enzyme activities. Insights into the distribution of adult female tissues, 8 *P. puparum* SPI genes (6 Kazals and 2 Pacifastins) were differentially expressed in the venom gland. Among them, 2 Kazals and 2 Pacifactins were detected in both of the venom gland transcriptome and the venom proteome (Supplementary Table S5). The other four Kazals were significantly expressed in the venom gland as well. However, we cannot identify these proteins in the proteomic data. These Kazals are small peptides with the average of the molecular mass at 8.6 kDa (Supplementary Table S1). These small peptides may not be retained by SDS-PAGE used for proteomic identification, and therefore missed in the proteomic data. Molecular and biological researches such as western blot are necessary to validate whether or not these Kazals were secreted into the hemocoel of the host. It was reported that venom proteins were recruited from non-venom proteins through several ways, including gene duplication^56^, alternative splicing^43^, single copy gene^57^ and lateral transferred gene^58^. Kazals and Pacifastins specifically expressed in venom gland may also be recruited from non-venom proteins through gene duplications and probably co-function in inhibiting host immune response.

All in all, the study provides us with detailed information of serine protease inhibitors in *P. puparum*, including gene structures, domain organizations and putative reactive sites. This information could be helpful in discovering the potential functions of *P. puparum* SPIs, which are crucial for wasps to keep the homeostasis themselves related to reproduction, development and immune response, as well as to evade their host immune system.

## Materials and Methods

### Insect rearing

*P. puparum* colony and its host *P. rapae* were reared under the cases of 14: 10 h (light: dark) photoperiod at 25 ± 1 °C^31^. Pupated *P. rapae* and mated female wasp of *P. puparum* were placed together in a plastic tube. Once oviposition was observed, the wasp was taken out of the tube. The parasitized pupae were then reared under the conditions mentioned above. Once the new wasp adults emerged from the parasitized *P. rapae* pupae, they were collected immediately, held in plastic finger-type containers and fed with 20% (v/v) honey solution.

### Identification of SP/SPH genes from *P. puparum* genome

We downloaded SPI genes from the genome or NCBI GeneBank of *D. melanogaster*, *M. sexta*, A. *mellifera*, *A. gambian*, *T. castaneum* and *B. mori* (https://www.ncbi.nlm.nih.gov/genome). These genes were used as queries against *P. puparum* genome database using local BLAST program (E-value 1e^−5^). Identified genes were validated manually using NCBI online BLASTP. The domain architectures of these SPI proteins were calculated by PROSITE (http://prosite.expasy.org/) and SMART (http://smart.embl.de/).Theoretical isoelectric point (pI) and molecular weight (Mw) of these SPI proteins were carried out by pI/Mw (http://web.expasy.org/compute_pi/). We also annotated the SPI genes of *N. vitripennis* genome^59^ in silico for comparative analysis.

### Sequence alignment and phylogenetic analysis

*P. puparum* SPI sequences were aligned with SPIs with known function or structure from other species. ClustalX 2.0^60^ was used in multiple sequence alignment. Phylogenetic tree was constructed using neighbor-joining method by MEGA5.10^61^ with 1000 bootstrap replicates.

### Samples collection, cDNA synthesis and qPCR analysis

All of the RNA samples used for cDNA synthesis were collected as previous described^34^. The different developmental stages (embryos, larvae, female and male pupae and adults), tissues (fat body, gut, ovary, venom gland and the remaining carcass of 2-day aged *P. puparum* female wasps) and immune challenged samples (PBS, Gram-positive bacterium *M. luteus*, Gram-negative bacterium *E. coil* and fungus *B. bassiana*) were obtained, washed, and then pooled into centrifuge tube with Trizol reagent (Invitrogen, USA), respectively. Any genomic DNA in these RNA samples was removed by adding RNase-free Dnase (Promega). The total RNA was obtained followed by the manufacturer’s instructions. PrimeScript™ One Step RT-PCR Kit (Takara, Japan) was used in cDNAs synthesis. Primers for target and control genes (Supplementary Table S6) were designed on website Primer 3^62^. The qPCR was conducted in 25 μl reaction mixture followed the protocol of SYBR Green Supermix Kits (Takara, Japan) on BIO-RAD CFX96™ Real-Time System. The qPCR reactions were set up as follows: enzyme activation at 95 °C for 30 secs, followed by 40 cycles with denaturation at 95 °C for 5 sec, annealing at 60 °C for 34 sec. Dissociation curves were checked at the end of PCR reactions. The relative expression profiles of SPI genes in different immune challenge stages took *P. puparum actin* 1 as an internal control. Developmental- and tissues-specific expressions were normalized to a reference gene (18 s rRNA). We calculated the relative expression levels in accordance with the 2^ΔΔCt^ method^63^. Error bars represent the means ± standard deviations from three biological replicates. The expression profiles of SPI genes in different development stages and tissues distributions were performed by one-way analysis of variance (ANOVA) and the expression profiles of SPI genes in immune responses were conducted by two-way ANOVA.

### Analyses of RNA-seq Data

RNAseq libraries from 6 developmental stage samples and 3 tissue distribution samples were previously completed in our laboratory. Various life stages include newly laid *P. puparum* embryos, larvae, female and male wasps in pupal stage and adult stage. For tissue analyses ovary, venom gland and carcass without venom gland were prepared from female adults of the wasps. We calculated the FPKM values of *P. puparum* SPI genes (Supplementary Table S4). The expression profiling of SPI genes at different development stages were visualized using the R statistical program version 3.1.3. Differentially expressed analyses between venom gland and carcass were conducted by R package DEGSeq v1.2.2^64^ (Supplementary Table S5). Benjamini & Hochberg method was used to adjust the *p*-values. Corrected *p*-value < 0.001, log_2_ (FPKM_VG/FPKM_Carcass) >1 and FPKM_VG (Venom gland) >10 were set as the threshold.

## Electronic supplementary material

Supplementary data
