# Supplementary material for: Identification and characterization of serine protease inhibitors in a parasitic wasp, Pteromalus puparum
Source: Sci Rep. 2017 Nov 16;7:15755. doi: 10.1038/s41598-017-16000-5 (PMC5691223; doi:10.1038/s41598-017-16000-5)
Supplement: Supplementary file 1 — Supplementary data [file 41598_2017_16000_MOESM1_ESM.pdf]

Identification and characterization of serine protease inhibitors in a parasitic wasp, *Pteromalus puparum*

Lei Yang[1], Yaotian Mei[1], Qi Fang[1], Jiale Wang[1], Zhichao Yan[1], Qisheng Song[2], Zhe Lin[3], Gongyin Ye[1]*

[1]State Key Laboratory of Rice Biology & Ministry of Agriculture Key Lab of Molecular Biology of Crop Pathogens and Insects, Institute of Insect Sciences, Zhejiang University, Hangzhou, 310058, China
[2]Division of Plant Sciences, College of Agriculture, Food and Natural Resources, University of Missouri, Columbia, Missouri, USA
[3]State Key Laboratory of Integrated Management of Pest Insects and Rodents, Institute of Zoology, Chinese Academy of Sciences, Beijing, 100101, China

*****Corresponding author: GY Ye, e-mail: chu@zju.edu.cn.

Supplementary data

Supplementary Table S1: Predictions of SPIs in *Pteromalus puparum*.

Gene Name	SPI domain	Gene ID	Number of Domain	Size(aa)	Signal P/TM[a]	Locus/ Orientation	Exon	pI/Mw
PpSPI1	serpin	PPU03966-RA	1	402	SP(1-19)	Scaf_0/+	8	5.19 / 44590.98
PpSPI2	serpin	PPU05464-RA	1	404	SP (1-23)	Scaf_2/-	8	6.39 / 46454.37
PpSPI3	serpin	PPU06944-RA	2	830	SP (1-24)	Scaf_4/+	13	8.66 / 91951.59
PpSPI4	serpin	PPU06883-RA	1	623	TM (28-47)	Scaf_4/+	8	7.36 / 70218.71
PpSPI5	serpin	PPU01309-RA	1	442	SP (1-24)	Scaf_180/+	4	6.34 / 49365.94
PpSPI6	serpin	PPU06845-RA	1	719	/	Scaf_4/-	9	7.75 / 78046.99
PpSPI7	serpin	PPU03962-RA	1	386	/	Scaf_0/+	6	5.95 / 43569.87
PpSPI8	serpin	PPU03364-RA	1	353	TM (37-59)	Scaf_1/+	5	8.77 / 40275.17

PpSPI9	serpin	PPU06096-RA	1	418	/	Scaf_5/+	7	8.82 / 48499.11
PpSPI10	serpin	PPU03963-RA	1	250	/	Scaf_0/+	6	6.60 / 28068.17
PpSPI11	Kazal	PPU00458-RA	1	972	SP (1-25)	Scaf_240/+	7	7.72 / 105848.31
PpSPI12	Kazal	PPU03796-RA	1	86	TM (26-48)	Scaf_0/+	4	9.10 / 9933.84
PpSPI13	Kazal	PPU05137-RA	3	442	SP (1-31)	Scaf_3/-	5	9.22 / 47457.62
PpSPI14	Kazal	PPU05579-RA	1	228	/	Scaf_2/+	3	9.31 / 25229.26
PpSPI15	Kazal	PPU07660-RA	1	98	/	Scaf_6/-	1	7.47 / 10883.32
PpSPI16	Kazal	PPU12052-RA	1	81	SP (1-24)	Scaf_111/-	3	7.61 / 9005.60
PpSPI17	Kazal	PPU12053-RA	1	130	SP (1-16)	Scaf_111/+	5	7.51 / 13963.67
PpSPI18	Kazal	PPU12054-RA	1	112	SP (1-20)	Scaf_111/+	5	8.61 / 12610.37
PpSPI19	Kazal	PPU12067-RA	1	115	SP (1-21)	Scaf_111/+	3	9.06 / 12249.48
PpSPI20	Kazal	PPU12265-RA	1	81	SP (1-23)	Scaf_17/+	3	6.52 / 8965.34
PpSPI21	Kazal	PPU12266-RA	1	75	SP (1-23)	Scaf_17/+	3	8.45 / 8263.74
PpSPI22	Kazal	PPU12267-RA	1	81	SP (1-23)	Scaf_17/+	3	8.12 / 9010.51
PpSPI23	Kazal	PPU12268-RA	1	84	SP (1-23)	Scaf_17/+	3	6.51 / 9233.73
PpSPI24	Kazal	PPU12269-RA	1	76	SP (1-23)	Scaf_17/+	3	4.83 / 8484.73
PpSPI25	Kazal	PPU13579-RA	1	79	SP (1-23)	Scaf_19/+	3	6.01 / 8483.85
PpSPI26	Kazal	PPU13580-RA	1	88	SP (1-20)	Scaf_19/+	3	6.16 / 9592.25
PpSPI27	Kazal	PPU13581-RA	11	895	SP (1-20)	Scaf_19/+	15	5.76 / 99226.37
PpSPI28	Kazal	PPU13582-RA	3	287	SP (1-17)	Scaf_19/+	5	5.75 / 33066.25
PpSPI29	Kazal	PPU13583-RA	1	77	SP (1-22)	Scaf_19/+	3	5.00 / 8301.57
PpSPI30	Kazal	PPU14014-RA	1	69	SP (1-19)	Scaf_18/-	2	4.75 / 7900.13
PpSPI31	Kazal	PPU14466-RA	8	496	SP (1-22)	Scaf_27/+	9	8.18 / 54825.71

PpSPI32	Kazal	PPU16162-RA	1	93	SP (1-18)	Scaf_553/+	3	6.01 / 10182.92
PpSPI33	Pacifastin	PPU02466-RA	1	184	SP (1-20) TM (55-74)	Scaf_166/+	9	5.34 / 20745.33
PpSPI34	Pacifastin	PPU03404-RA	1	298	SP (1-19)	Scaf_1/-	8	5.13 / 33002.73
PpSPI35	Pacifastin	PPU07367-RA	7	342	SP (1-16)	Scaf_7/-	7	8.32 / 37181.39
PpSPI36	Pacifastin	PPU07368-RA	8	548	SP (1-17)	Scaf_7/-	9	5.93 / 60730.04
PpSPI37	Pacifastin	PPU07369-RA	5	296	SP (1-17)	Scaf_7/-	4	5.72 / 32891.42
PpSPI38	Pacifastin	PPU07370-RA	9	566	SP (1-16)	Scaf_7/+	6	7.00 / 62798.29
PpSPI39	Pacifastin	PPU07670-RA	4	407	SP (1-19)	Scaf_6/+	11	7.93 / 44040.10
PpSPI40	Pacifastin	PPU07672-RA	2	239	/	Scaf_6/+	6	4.87 / 26396.84
PpSPI41	Pacifastin	PPU07674-RA	1	84	SP (1-20)	Scaf_6/+	2	8.97 / 9751.43
PpSPI42	Pacifastin	PPU08700-RA	1	143	/	Scaf_246/+	4	4.47 / 15991.36
PpSPI43	Pacifastin	PPU10736-RA	2	372	SP (1-23)	Scaf_21/+	6	7.97 / 41625.72
PpSPI44	Pacifastin	PPU14859-RA	1	73	SP (1-26)	Scaf_118/-	2	8.17 / 8193.63
PpSPI45	Pacifastin	PPU09199-RA	2	299	SP (1-19)	Scaf_158/-	8	5.13 / 33076.76
PpSPI46	TIL	PPU14671-RA	1	82	SP (1-23)	Scaf_119/-	3	6.02 / 8786.24
PpSPI47	TIL	PPU14688-RA	1	82	SP (1-23)	Scaf_119/+	3	7.42 / 8962.53
PpSPI48	TIL	PPU14689-RA	1	88	SP (1-24)	Scaf_119/+	3	8.28 / 9542.35
PpSPI49	TIL	PPU14690-RA	1	85	SP (1-23)	Scaf_119/+	3	8.49 / 9258.00
PpSPI50	TIL	PPU14738-RA	1	83	SP (1-24)	Scaf_119/+	3	6.77 / 9125.82
PpSPI51	Kunitz_BPTI	PPU01181-RA	1	922	SP (1-22)	Scaf_186/-	15	6.15/105161.10
PpSPI52	Kunitz_BPTI	PPU14032-RA	1	2035	SP (1-25) TM	Scaf_692/-	25	5.45 /

					(1761-1783)			227444.34
PpSPI53	Kunitz_BPTI	PPU14848-RA	1	790	SP (1-28)	Scaf_118/+	13	6.34 / 88680.14
PpSPI54	Kunitz_BPTI/WAP	PPU12795-RA	11/1	2827	/	Scaf_16/-	39	4.65 / 309067.31
PpSPI55	A2M	PPU07478-RA	1	1887	SP (1-19)	Scaf_6/-	20	5.87 / 208361.87
PpSPI56	A2M	PPU08491-RA	1	1702	SP (1-38) TM (1672-1694)	Scaf_8/-	13	6.21 / 195426.92
PpSPI57	A2M	PPU09870-RA	1	1450	SP (1-18)	Scaf_35/+	23	5.74 / 162760.09

[a] SP, signal peptide; TM, transmembrane region; /, neither signal peptide nor transmembrane region detected or incomplete gene (missing some exons or 5’ terminus and 3’ terminus).

Supplementary Table S2: The predicted amino acid sequences of 57 *Pteromalus puparum* serine protease inhibitors.

Gene Name	Gene ID	Length	Sequence
PpSPI1	PPU03966-RA	402	MRTPTVILALALCVTCTMAEDKAVEALKAVSEGTQYFATNFFKQVAAENKGKNLISSPLSAHVVLSMAAFGAGGNTAVQMRQSLHMPADDVVSKQGFENLIDTLNNVENVTLEVANKMYLANDLKLKSDYKQLTSGTFRSEASEIDTKKPAESAKLVNDWVKEKTHNKIEDIVNEDDITTDTRMLLLNAVYFKGKWAKEFKKEGTQDKIFHLDAKTEKKVPTMFASGSYVYGELPDLKAKFVELPYENKDLKMVIIVPDEIEGLSAIQENLESFNHTRLAEAGSERDVQIYLPKFKIESTIDLQKPLEALGMTDMFTNSANFSGVSDELLKVSKVTQKAFIEVNEEGSEAAAVTVINYFVRSGPRAIAAKFLADRPFFFMIYDIKANIPIFTGSIADPSNEL
PpSPI2	PPU05464-RA	404	MEIRFKGIYIFIFQLVISTGVNMDPELNFSYISNSCSDFTSNLFQVLTSEQHMENVASSSLSTYILLSLLLHGTNGETREEIKSGLNLIDVDKTQEELQSLFMQLNNVTDADLQLANGIYVNSNFQLQDDFMSKCQKYYQTSIEKMISQNSDHAAKQINAWIKEQTKNKILNIINSDDIDEDTKVILINALYFKSSWLNPFESQFTKKRKFYSIDGTETHVPTMYKTFNVLHGYIKSLRSRFIKMKYLSKEFEMILILPDEKYGLHELEKNFDWNKISKASCFTTEVELFLPKFKVEATMNLESALVKLGIKTMFTERANFSLMAKTPLHVDRVFQKIIIEVDEKGSEAAAATVAQIRARRGISSTDVFAVNHPFMFMIHHRPSSIPLFIGSIRKLGGNYKDEL
PpSPI3	PPU06944-RA	830	MSTILRAGWIALMILIIGMINVTAKTNGTSQNDIDDFVPYQGERSNIFDWNLLQNLAKSHRGNLLISPISLKLALVLLYEGAQDETAQQLATVMHLPVGILATRDKFSSVLKSLQTKRPEYVLNVGTRIFIDQSITPRQRYGAILKSFYNTDVLNVKFSDTKSSAELINNYVRNITEGRVQKLVEDESKLRNNMLLIASAMYFQGTWHRQPFSANQTQIGKFRLGDGKSTIQVPFMRSSSKFYYAFASDLDAKILRLPYAGHKLAMYVILPQAPGKLDELVKKVSPFVINRHVWLMQEIYVDVMIPKFKFDFTSKLENNLRELGIRDIFDDTATLTGILRSKSTSRRLVVTDIIQKTGIEVSERGTVAYAATEVDIGNKMNDETFQADQPFLFYIEDESTGTILYMGLVNNPLEETGISESSAAATSQSPQMPSRVGGHEDAPTASPAIGTGIDERQNFFNVELLQALNEAKPGNVVVGTSSVKAALMILAEAAAGRTRQQIVSTLRLPTDVAQIRDVVSHSISSFKDPKSDTQLQTAIKVWLSKNVALHKDYTDILQRYYKGELQATNFADVAGTVKIINDWAKKCTNGHISSILEPNSVAADTKMVLTTAVYFKGTWLNSFDKTATRSRCFNVPKLGCQQVPLMEVVGNYKYGYVPALDAQVIQIPYTGKRVSMVVLLPQRLGEQALGDLSRDLAFTPMSVLLSSL

			QETEVLLQLPRFSIGNKVDLRAALEKLGIKDLFDKNANLTTAFPLANVQVGAIMHNAQIEVNEEGTIAAA<br>VSGVSVIPLMGSTSTTFRADRPFLFFLVDHQTNSILFAGRYFQPEGPTTKTV
PpSPI4	PPU06883-RA	623	MTHYNTSRKSFIRHDDRPKHQPKIRMKFLLSMSLLALVAVSYAQFIYPDEFESLREEEQRRQNTAGGLHLP<br>TFEPTVQSINDVESAPYYPPPLGQSGFQQHDQPASRPPAAFAPVGVNGKPTNNPFDTFLPPRWRDHVINIIS<br>RGVTKFTLDMDRAIEKSSPANSRENLLFSPVSLTLTLAMVMLASNGKTFEEVTKILGLESGVDISHHSEIVH<br>QIFGLLIQQSEYMQYLDPSAPQCKLAFGIFVEDGYPVREQFRAVSEKVYKSEVISVDFSHHAKQAQSVIND<br>WVSNKTNHKIRNMLYEPPNPLTDVIITSALYFSGEWEQHFMEGSTKRKPFTIENGETVYVDMMYNGGYFP<br>FYEDKELGVKIIGFPYKGQEVTMYAILPNNPGAAALSEMKHRLTPEIVDNLVANMKNSSCIIGYPKMKLSS<br>TLKLQSALEALGLSSLFNPHTADLSVLSPGRAGPNRTQPPPPPSTPSRAPATGFNGGFHQPTKTLHSDYTFE<br>PRFHGGRNNMYRRAKRQSRPIDQEFVDFLNSQKLPTFGVDELRNSAGISNPGLYADDVIHKVEMTVNEK<br>GTEAAAATSVILDRSGDYKRFIANRPFLFFIRHDLAKAIWFWGTMNRPTPFYETP
PpSPI5	PPU01309-RA	442	MASSITKMSVALLLLVSIFSQTHSQCLTGNDNPSTMRQDAAQLLSDARFDFALESLKKIAEIETQDNVFFSP<br>HSLHEALGLAYFGSRGTTEAALRKALHVPQDFSKVDVQRFYAFEKSLEAARKANSSANYDYRVANRLW<br>LSGAKKLRDCMLDFFGQELQRVDFKANPEAVRKQINDWVSDQTRGNIRDLLPASAVDESTDAVLANAV<br>YFKGLWQSKFLPENTKRDVFYLGQDNMTIAQFMKQKGSFNHMVSEELGVHILQLPYKGDDVSMYILLPP<br>FVSTQQVAQRSASQPKSDGVRQLLQRLSDNSDSAKELRDILDNGMPARDVELAIPKFSLERELPVKDLLV<br>AMDAGVVFDTSSDFTGFVADGEKGIHLGDAVHRAKIEVTEEGTTAAAATALFSFRSSRPTEPAFFTANHPF<br>AYFIYDRPSRTVLFAGIFRKPNKK

PpSPI6	PPU06845-RA	719	MVGYVSWVCGVFVAGCILQPPSPPVNAHYHDLYHQQHFQPWHRHRPAPIPRRPVDLMTDVVNDLGTRIL QQYVEPGNVAFSPAGMGFILAALYEGSTGHSRQQIVDCLGLPRDRNTVRVGMRDIHRRLRTYLNPDGFL GGLNLNRENTTLRPDYENILRFYGFDLSIDLSNFTADNTGNFGLRGSSTTNMPPMTSQTQMATTPPPGSTM MPNGAATGQTLPPGTTMMLNGAAAGQTLPPTTIPPAMNGGMAAGTTMPPTTQAPAGTTTIDTSVVLISTT QAGAPITMPPAAQTATTGAPTTTMATGGAQAAATIMTSTLATTTTLQPTTMPPTTTMAANAALISTTLPR LPPSTPGNRAFLTAGETTTTDLPTTTTTMPTTTTMQSSGIGAETTTIAEDIVTVAVPTIIPALIRRRRSRRRRR SNEGYFSNYPDDGLWMQDLDIWADQPVLPTNVRDATELQFLVNGCDLATVPAATYTTVLPFAYFPSLKA VALEFPLDNPRYNVLLFMPTERIDTSRLSRELAGQNLRLLRRQLQPTWLRATIPSFMLRGFVTLTPYLQRL GIRDVFEPRMADLGPMTPDLGVYARDVQQSIAVNIRNYMKPDPNGMNVNPSNNPNINMRPNPPNMAMG QNGNMPGAPGGGQMPVPRPLRPEDPPVYVPPPRDSYRYTRPVNGVDSEHPSIVPFTAEHPFLFFIIDSETSV SLIAGRIDDPLNSRIL
PpSPI7	PPU03962-RA	386	MAENKHEEAIAHVAKSAQSFTNDFHKKVAGETDGNFVSSALSAHVVLAMAAYGADGKTKEEMRQTLH LPEKDEVAHEGFQHFIHAINNVPDVILKIANKIYGANDLKIKDRFLEITGKHFHSECSKLDFSKAKESADEV NNWCVEKTNGKIKDLLTESDITSDTRMILLNAVYFKGKWLHKFHEERTELKPFHVNKTTTIDVPTMHITK KFFYKDLKELNAEVVALPYENEDLALVIVVPKEIDGLKQLEDNLDKIQIDEHDLKRYKREINLALPKFKIE TTIDLNKHLDELGMSTMFTNNADFSGVASEPLKVSKVLQKAFIEVNEEGSEAAAVTAVQMVLRCAVFDP TPPLVLNVDKPFVYKIVYKGNPLFSGHIVNPLEQTSK
PpSPI8	PPU03963-RA	250	MNFTFGSFDWSQTFKDVYVELVPYPNVRKSTQSFNTDFHKNVANTFHNANFVSSSLSAHVVLTMAAYG ADGTNKAEMKTSKATGKVGARMVAIPYKNPELTMVIILPNQIDGLRKIEDNLDKVNYDSSEFINKTEVILA LPKFRIENTIDLNEILQRLGMTQMFWNGHNFPGISDEPLKVDQVIHKAFIEVNEKGSEAAVVTAVMTSFGS TMPSQPKPPPIEFIVDRPFLYKIVWKGVPLFSGHVYNPIN
PpSPI9	PPU03364-RA	353	MMREKKCQDAITQVAKSARSFTNNFHKKVASKTDGNFVCSAWSAHVVLAMATYGAGATGRTKEQMR QTLHLPEKDKVARKGFYYLIRAINRVPNVIFKIANKIYGANDLTIKERFLEITGKYFHSECSKLDFNKTKES VDEVNNWCVEKTNGKIRDLLTEADITPDTRMILLNAVYFKGKWLHRFRETATESKSFHVNKTTTIDVPTM HITEEFFYKDLKELNAEVVALPYENENVALVIVVPKEIDGLKRIEDNLEKIQIEEHNLKHDKREINLALPKF KIETTINLNKHLNDMGMSTMFTNHADFSGVANEFLKVSKVVQKAFIEVNEEGTEAAAAVTGKQSISNLV

			YDRS
PpSPI10	PPU06096-RA	418	MSHPSALYHRIIINRDIYDQYEHRNTRTFPRFVKGIEKFTNDFHKSISNDLEGNFVSSALSAHVVLSMCAYGAKEKTAEEMKRALCLPTSVCWESFRCLLKTIDDSSEIGVATKILIDKNIVVKDSFKQAVESFFYSEITEVDFVNDSRNIIKDVNKWCLEKTHHKIREIISPKEPLEPTAKLMLLNVIYFKAYWQNKFQPACTFMDEFHIDAKKTIQVHMMHQLEKMHFKELNAVDAECVGIPYKNSDFVFVIIKPNKIDGLKFIENNLEKINLNYREDFKIDSFKDYGKGHTHTNKNIFYVNLSLPKFKIETTIDLEKNLKKLGMSTMFTHRANFKDISTKPVLVPRLHVHDVIQKAFIVVNEEGTEAVAMTRLNQCGGGSIEIREFKVDRPFLYRIVHKSTNVTLFSGCVSRPKY
PpSPI11	PPU00458-RA	972	MQRLSMIIILLLSLLLLLLLRDGVAEELSCCAVATGSCRSVCSKVSLLSLAADSYARENATRIFTEFCPPESVEFWDCVNSTLRELERNENWTGRSCCHLARSLTCRSACATAGHQRDLKLSCRWSDEFALSDCLEHREEVEKCCSSVSNSSCRSICKELFHKSGGRRSALKMYKSKGCFHQVPKCLKGLVDDSSKPSEDPKLLADCCEKAPNRGCVESCLNLIHTLGSIDEVLEGLDNSTPSCHPVKPHSPPWSCFLSRSSPSSTGTSKNRRLPLNVAKLSCCSRAKRKTCRSLCLRAFQSDWSAWQQLENQCLSSSPALETELSRCLDDSEDSCEMGCSGLSFCSKFNDRPTTLFRSCTRAADEAARWEADHWIRGGVIAGLGVPVRAAPSCPAETLRAAACLLQLRPCEARAHETRLCREDCMDLMTSCVDWSAVRGHNAATLCAKLSPPKPEQPCISIKPFFELDNEPPRRTDIDEDINMPCRSNPCVQGQLCVVQPEERRGYRCVPGCTLGAMSNQLIPLSSWAQLSVPKHQKQEQQLCQSICQCVISDDPGVSGPRLERCSEPSNCRVAESGCSLQTLNSMIVAHDFDFYLECNSCHCYDGEITCSRRSCAEPAAASLPCNCPQHYVPVCSRLGVTYASACLAKCSGLLANEVEYNSCSARDPCAARPCGPGYTCLPRPRVCLTAPNHRPCEQFECVRLSPSSCAGHAHQKPVCDSENRQHSSVCAMLRSGARLGYRGPCLRGCSLRGPVCGINGETYASECAAWAERSLVDYQGPCLAVGLVSEQPRPRCGESVQCPVLSRSSCLGVTPPGACCPVCAGAARLFFSRKQLDLIFYQLPDSDSEDKQPVTLEAMLAALSRQLQVAECALRGSLTPEGDIFIVNQPISKRPSNLQLAACITEIEKLVSRIQARSPRIVNEVPLSALTKAEVAHVRIVGNAATAAATANSVTSLATLLIILSLLNLAR

PpSPI12	PPU03796-RA	86	MLILFLQTSNSLRRVIKQTFKMNKQLVFFFFIVMIAMAFGCICPRNYQPVCDNLGKQHNNLCLFNCAAEQ AMRNGQELTIVKHGEC
PpSPI13	PPU05137-RA	442	MTGELRARATTGAALLLPLIIALLRARPATGGACWSNVNSAGRCKEILSQGVSKEECCGGANAPETTAYS EENYDNGALFFWQVLGGGVKCDSCRSSCLGVSCSEGRKCVLRRGMPKCVCRPECREIKAQAEGPVCGTD GRTYRNVCKLKRRVCRKGYHELAVAYGGQCRSSCLGVRCRHGRSCLLDQNLSAHCVKCSRRCSIGSASD QPQQQQQQQQQQPPQQGREPLRPVCGVDGNTYKSACHLRAAACRAGRAIAIAYKGPCRKYTDCRSIQC RPGQLCLTEPHTERPRCVTCLYRCPRNKELVRERNRHRNRDYQDPSPIALCATNNVTYPSWCHIMKDACL TGLVLETRHAGACNAQDPAPFHENASASKELQQLSKKPIAGKGKSSKSSAASPAASSSSSSSSSTSTSTST SSSTSGSTSSSTAADADTSSSFL
PpSPI14	PPU05579-RA	228	MKTFLAILVAWNGEFKFGNYRKRSTLNVTVFSTTLAITNCVCDNRTVPATVSPVCASDRKTYCDAQLVA CMNTCYQTSRLSHYRVFNLSLLRTLNVPDLRIVSYGACVATTRRPLGTLPTIVPLYPTATLAPILNPNAAG RCYCPNTGAYSPVCVSDGRTYANAGLLACYNRCYNRNLSVIRLGCCPRNLCICTNTNYHSPVCGNDRQT YKNPKIVQCLNTCQNRSML
PpSPI15	PPU07660-RA	98	MYTQEKKSKPTQTYEPLPHLLPEPCEITFCGWGMSCVISESGKAMCQCPSGCPESYSPVCGDDGVTYDND CQLRRASCQKRKDTRVKHQGACGKSQQQ
PpSPI16	PPU12052-RA	81	MLKTIALLLLLTVSAFYLWKGIEAMQKNGIKCGCRIPRIYRPVCGEDNVTYDNAGVVRCINICNHTDLKV LHEGVCAGDDH
PpSPI17	PPU12053-RA	130	MRCVLLVILAVGFCHAQIDQFSNQDGFVFPGEADRPSKFPLNPTINPNRPFTTSSQRTTVSPTSAVNPVTPN LADYNQCINNCQTTSEYNPVCGTDNVVYSNPGRLNCAVTCGNNIAIKGYGQCSGSIRG
PpSPI18	PPU12054-RA	112	MTRQQFVFATILLVSSRIYAMPQDIVFPGETFPFPTTTEASTESTSISWTTVSLPSNYAACLHNCPTISSYQPV CGTDGVVYQNIYKLQCANRCGNDVRVFKEGQCRLRRRQ
PpSPI19	PPU12067-RA	115	MKLAFAIGAILLVCLVCSIEAGPTEKPATTVKPTASKEVKEAIKNAKEAKKAKKDCLKSCPTNYVPICAHD PANASFKPRTFGNQCVLDTHNCEMGTKLVVKMKGECPGSDGVRL
PpSPI20	PPU12265-RA	81	MFKQIVCLTLCVLLLAMIANTEAEGEPKHCACKITKEYKPICGTDNQTYDNWRKIACKNKCQGTNITVNY NGVCAGDTSDD
PpSPI21	PPU12266-RA	75	MFKQTVCLVLCVLLVAMIANTEAEGEPTRCACKVTRIRNPVCGSDNKTYDNISKLTCKNKCEGTKLIVKY

			DGECS
PpSPI22	PPU12267-RA	81	MLKQIVCLTLCVLLVTMIAKTEAEDEPKRCACKVTKEKKLVCGSDNKTYSNWRKLACKNKCDGTNVTVVHNGQCEEDTSEA
PpSPI23	PPU12268-RA	84	MARIDIFVVLCVSLMCCTLLVDATVIRFRNRQEAIANNKCDCISAFEFKPVCGSDGRNYPNVATLNCANECQRTSIGISHDGLC
PpSPI24	PPU12269-RA	76	MAKSMLIVAFLLVACMMFMHVSAFTDQDGNNCPCATTDDLRYVCGSDGVTYDNESELNCENKCKRSNIQVRFEGKC
PpSPI25	PPU13579-RA	79	MSKAFVILVSSIVILALAIESRAQETQVLPEGCVCAATDELDPVCGNNGVTYPNLATLKCANECVKYSIHHMHHGPCRT
PpSPI26	PPU13580-RA	88	MMKQVVIFVAMAMLIAFVQGQDENEGLKDCDCITTFEYMPLCASNGVTYSNPGMLECAKKCLGKSDLSKVRDGACLQTKETPAKTTTK
PpSPI27	PPU13581-RA	895	MMTYYLSCIILIALIPSAILQENLDLKYELFNIDSLKDCRCNCTLDLVPICKNKDVTYDKKEMSDCINKCSKNSTLNIIHNRECSEKSVNQLPSKPTKECKDCKLTNEFSPVCGNDGVTYPNPSYLFCVAECEKPGLLFRHCGFCLSQDKKKFEDNEVIMNDDSKKVSKDLLDSLATCDCVIKYNLNPICGSDDLTYPNPSMLYCIKKCRKPDLQIKSCGKCPAKAPQETTESKIAPKCTDAINQCLASCAVTADINPICGTDNKTYVNISVLKCHNTCTNETINVKYYGTCDEKCACVADKEYNPVCGSDGNTYSNPSTLFCAEKCIKPDLKLSYCGKCVEDQEELVTPQKEAVIPDELAKMFETCGCIFTKEYAPLCGSDGTVYVNPSIFYCARKCIQSDIKIKNCGECYKINQESNVKTFQQTVSKNFKSLSSPFLKCDCVATSTLYPVCGTDGVTYPHPSYIYCTKNCKANDASIEDLQMAYCGPCKEGGQEIPKSDKTQLSSESLDMFEKCKCKVTVESNPVCGTNNVTYGNPSILYCSNKCIDPTIHIKYCGICREKDNPKPLNVNDFDNDDNDPNKCRCTATREYKPVCGSDGNNYANFVVLECIKNCTNQNLNVAHDGLCSDENKDSFITPKRNARSAQVSGSSQGIDALLGCNCYVKTNYAPVCGTDGKTYPNLSQLSCNNWCYRRYVYIDHYGPCNDYTRPALVSKSCRCAVNYSYKPVCGSDGETYPNDQQLDCAISCKNNKNLYKMHDGPCSEAAKAEEPSTPQPSPQSCQCSFTYEYAPLCGSDGEDYVNPSYLRCFNYCNNTNVYKAHDGQCQYPTKYGDPPISKRCACVHTYDNAPLCASDGKTYGNLSQMNCANSCYNQNIHKLYNGPCFDEAISAAAA

PpSPI28	PPU13582-RA	287	MKLKVFFFVFTLTYVDAQLLLLPPISEAVLSENSYECSCDFIYDEYDPVCGSNGLTYSNPSYFYCDQKCKD SNLSVFRNGACSEDTTNLPTSTNDSWSPYERIKNFNRCNCPKSVEYNPVCGTNGITYDNPAQLQCEKKCR QPQLNIARYGFCREEEYFKDPLPFFTDAIKRCDCIYRNERDLVCATDGYTYPNPSTVSCVTRCNNPSLRIVH YGRCKENQKPHNYGAVKCKCTPSSEHYQPICADNGITYDNLSLLYCARECLNRNLKIVSEYPCRDYLNVD ERKI
PpSPI29	PPU13583-RA	77	MKVILFSVLIVVFASCFLQGEAAPQYDQGVDCPCAATANIYYVCGSNGVTYTNPSLLRCAEKCTDTTITKI HDGKCE
PpSPI30	PPU14014-RA	69	MNKRLISVFFIVMIAMAFGCEEEQCQKVYNPVCDNLGNTHINPCLFRCAAEDYKAENGTELTIVKYEEC
PpSPI31	PPU14466-RA	496	MRNCYFVIGIVLLCCIQEYASSSCPRICPTSGEPVCGSDGVIYASSCEMRKKTCGKGVSVATEKTACLRSSS SKCEHRCPGDQDPVCGTDGRTYLNKCMLRVEICRVGIELSHLGPCNNISAHRENCPVSCEQAPLDGPVCG SDGNVYKSTCQMKLLTCGQGVVRTNKKHCQTTRHCRESCWRGAKPACGSDGILYANTCKMRAKNCGK HVFEVPMSFCVSRERTSGSAATACPLDCKNEPEVAVCGSDGSIYRNECEMQMLNCGNTRRKVTAVDFEK CRNRLSKCTKQQQHCGTEVDPVCGSDANTYPNQCHLNVAICMKGIQLAHVGECTTLKETEHCPEDCNDV PEEPVCGSDGNVYRSLCQLQKETCGQRVVQVPAQHCRTTALCNQICSGERQFVCGSDNKLYRNECEMKR DNCGKHVYVVPMKRCVQGFMFRGCQKICPPYYDPVCGTDGMTYSNECFLEIENCRTRNHVTKKYHGLC GQPTEEPKNYLY
PpSPI32	PPU16162-RA	93	MLNVLFVVLALIASFCTSARIFTIVNTDEPVPDAKSCIAQRCANVPDLVPVCASNGETYANLHSLICINKCN KSGIQFVHYGLCDEKFKDVIF
PpSPI33	PPU02466-RA	184	MSKILKVALLLLLVAVAVSSYSVQDKDDDSNLPHIDDYNETNKCPPNQRFMWKSYVFVMYFSTFFISDIY SSSAQSVPGRDDWYKTGLKYDDLPRVFSLKNGCPAKEFYLDCNKCSCVGDSSDPACTYKACPQPPQIVN WKTGKSCPAGQHFYWKCNDCNCAENGREASCTRNFCPDFGDSSSE
PpSPI34	PPU03404-RA	298	MRESILILTIATLGIAAQAKFMTFGDTPTLKCVPDSQFTYDHNMCYCNKDGTELTCERKMYSTLTPELHLI QLQNLTLDCLPERHFKLFNQDCICHESGNFASCVGSQYGGIDSREKCIPGAVFFEDNCNGCICGTDGKATC TKLDCNIIGSYSEEPKATDLQCVPGSLAVFGCNDCTCVNSGTFLMCNKMGCGSMSLVHEHVLNVTMDCQ ANSIFDYNCHQCICDTKGNYAMCSGKECPHRYNLKKIKDTVKKCNPGMIFGSDCNICICAENGQGVCTTF SCDTTYQFKYVDDEWWL

PpSPI35	PPU07367-RA	342	MKSLFLVFLAVSAVAASESLYCTPGASFKMDCNTCSCSSDGKSAACTDMHCPEGNANVNDIDTIRAQPLC EPGKRFKLDGCSSCICSGDGTTAKCTLGFCANFKTRMAPRKSQYCEPGKMFSPDNCNLCKCSNDGTKAIC TMKLCEERKSRSANDQYCEAGKMFSPDNCNLCKCSNDGTKAICTQKLCEETEVTKQVCQPRSQFKDYCN TCTCSDDGSSYACTRMYCDKDVWNRDGSLKILVRNSVVLPERVCQPGKAYSPDGCNTCICNRYGTGQAC TSKLCLSNLKAAYQSVDSKKWGPLYKEGDACTPGRAFYSECNKCVCTRSGRSAFCTLMSCQTSP
PpSPI36	PPU07368-RA	548	MKTLLIFVVSTIALVSSEFHCTPGSTFQMDCNSCTCSNDGKTAMCTGVACIQENKDDVTDTDEKAETVAP VQTQGVGQAEFHCTPGSNFHQDCNSCICLKDGQSAMCTGIACPKKVKRDIETGPQQVCVPKSQFNNYCN TCGCADDGLSFICTRRMCDPEIWNKDGTMKISPKSLQRAARSIISDHKCKPGHLFKNDCNHCICDADDNTI LYSASYVPNNASILFDKTRVVSGDLDCEVDRAVIRRNSKVPINMKPIFVVLLTISVASARNIFSCLPGSVFM WDCNECTCSNDGLSAACTDMACPGDLNELSVYQPVLLKQQKVCEPSTAFRVYCNTCSCSIDGSWFACTQ MACDTEIWNVDGSLKVKSTAVRAKRSLASQEKVCEPRTQFKEYCNTCGCADDGLSYICTRRMCDKNIW NKDGSLKINITKDVVKRSAPQQICEPHSHFKNYCNTCFCNNDGSNYSCTRMMCPPEIWNKDGSLKIEYVR LEKSTEPKQVCEPRSHFKDYCNTCACSEDGTTYGCTIMMCDENVWNKDGTRKTEDAEKN
PpSPI37	PPU07369-RA	296	MKSLFVVLLSIISAVSARNIFSCLPGSVFMQDCNACTCSNDGLSAACTDMACPGDLNELSVYQPVLLQQQ KVCEPSTAFKVYCNTCSCSSDGSWFSCTRMACNSDIWNIDGSLKVESTAVRTKRSLASQEKVCEPGTQFK DYCNTCGCADDGLSYICTRRMCDKNIWNKDGSLKIDITKDVVKRSAPKQICEPHSNFKDYCNTCFCNND GTEFACTRMMCPPEIWNKDGSLKIQHLQSRTVAEPQQVCEPRSHFKDYCNTCACSEDGTKYGCTIMMCD ENVWNKDGTRKIVDGEKN
PpSPI38	PPU07370-RA	566	MNTLFVVALAITAVAAENLCTPGSYFKKDCNMCTCSMDGKTAACTDMLCPGEMKNYAGIDNERFCEPG KKFKIDDLSTCTCSADGTKAKCTLGVVSLFGKMMSSGQSQYCTPGKIFSPDNCNTCKCSADGLKAMCTL KICSDDNILDVKSSSTKQVCEPLKQFKDYCNTCFCSNDGLSFACTRMMCDKAIWNKDGSMKIVATTYQN QKKVCQAGSRFNDYCNTCFCNNDGTDFACTRMQCDKDLWNKDGSVKVFATTYQNEKKVCQAGSRFNN YCNTCFCNEDGTDFACTRMQCDKDVWNKDGSMKVRATTYDNKKKVCQAGSRFNDYCNTCFCNEDGT DFACTRMQCDKDVWNKDGSVKVRATTYDNKKKVCQAGSRFNDYCNTCFCNEDGTSFACTRMMCDESI WNKDGSLKIQAVEPRVCEPNTHFKEYCNTCACSADGSNKACTMMECDLNIWNKDGSRKDVLDNKRASD KVCEAGKVFSPDGCNTCVCNEYGTQLACTSKLCMTTLKQAYEVDETREWGPLYRQGDACTPGKPFYSE

			CNKCVCLETGNRAFCTLMDCAALA
PpSPI39	PPU07670-RA	407	MKPFGLAIISLALVVAASASPLLNSNSKTNVVDIPVFADYKPGDKCPAPVFNDGCNTCLCTADGVTAACTEMFCVNVNGQKRAVRSEEIPVFPDHKAGDKCPAKYFNDGCNNCVCGLDGVTAACTLMACLNFDGQKRAVRSEEIPVFPTYKVGDKCPAPVFNDGCNSCVCSNDGVTATCTFKLCLEQRQTRANVENLPVVYGYKNGDKCPAKHFYDDCNRCVCSLDGHSAGCTRMACPPNFGPRSVDTSVDVPVVSGYRQGYKCPAKHFFVECNACVCAENGYSAACTKMACLVPNPSDLFKIWMEKIFFPNVDSPKRCIPGSLVKDKCNGCICGPDGKAACTNLDCFTLIGQRSINLMVKKMISRAYQVIIWLLDVTNVAVIVMEQALFVIKLVANIQTSHLNYMF
PpSPI40	PPU07672-RA	239	MYSTLTPELHLIQLQNLTLNCLPESHFKHFNQDCICHESGNFASCVGSQYGGKDSREKCTPGAVFFEDNCNGCICGTDGKATCTKLDCNIIGSYSEEPKATDLQCVPGSLLVFGCNDCTCDDSTSMLCNRMGCGSMSLVHEHVLNVTMDCQANSIFDYNCHQCICDTKGNYAMCSGKECPHRYNLKKVKDTVEKCNPGMIFGSDCNICICAENGQGVCTTFSCDTTYQFKYVVDDEWWL
PpSPI41	PPU07674-RA	84	MHFTSTFFLLIFAIVSVSYANDAEMQCKPNTRFKYYCNICWCSEEGTIRICTKKYCPDNVFNKDGTLKTLPNVHQKLKIFPKGV
PpSPI42	PPU08700-RA	143	MMAITYAACTFMQCFDLDFEEEQRSKRSDEIVTKLSSDIPRISGYTQETKCPSKSFYNDCNICVCGPDDASAACTMMMCLPDETQQPPKIVPAKLTDIARIDNYMHGEPCPANQMFHNQCNMCMCSPDGYSAACTMMLCLPEN

PpSPI43	PPU10736-RA	372	MSVHHRLFSSVQLLFIFFAYVSTAVVRFGNEGDMICIPNATFHIDCNRCKCSPTGMSIDCTDWTCATLQIPHDLLLNLTLDCHPGEIFLYNYHKCLCTGSKQAMCTSAHNELAHNLRPTTGSIISSGNSPGKCTPGLVFKNKCDTCICGDNTRAACLGTGCIRHADRRRFEDLDFTGNYPHRKSTSVKLCVPDSIFSVDCNQCVCSDDGLSMSCDSYPCLGVEMRDEQMLNITFQCEANSSFLYNGYECLCSSRGKTALCSLMQYDKSKEFREAGKKVYAIADFTELCTPGMVFKDRCNVCICGTDEKAACVRIDCDLSWKYSRASEEKKGSGITERIIPKGEIWTQRLKGSTRKFCRRNGASMYCRYLRDF
PpSPI44	PPU14859-RA	73	MASKTTCVLLMSLLIIATFYVSVTEAKKQCVPGKYYFDGCNTCSCSETHVVYCTRKLCPDPWKPLSPPADFYQ
PpSPI45	PPU09199-RA	299	MRKSILILTIATLGIAAQAKFITFGDTPTLKCVPYSLFMYDHNTCYCNNDGTELTCERKMYSTLTPELHLIQLQNLTLDCLPERHFKLFNQDCICHESGNFASCVGSKYGGKDSREQCIPGAVFFEDNCNGCICGTDGKATCTKLDCDIIGSYSEEPKATDLQCVPGSFPVFGCNDCTCVNSGNLLMCNKLGCGSMSLVHEHVLNVTMNCQANSIFDYNCHQCICDTKGNYAICSGKECPRSYNFKKVKDTVEKCNPGMIFGSDCNVCICAENGQGVCTTFSCDTTYQFKYVVDDEWWL
PpSPI46	PPU14671-RA	82	MSKSSVLFFLVLCVGVSLLAVHASGSHCPKANQEWTGCGSACPSRCGQEEPRICTMQCIIGCQCKQGFMVRNDGECVLPEDC
PpSPI47	PPU14688-RA	82	MSKFLVLCLLVLCISASLIAVDGASSRRCLKRNQQWNNCGTHCPQKCGDPEIRPCTFQCEIGCECVPGTVLKGNECVDPKEC
PpSPI48	PPU14689-RA	88	MSKSIILCLLVLCISASLIAVGGASSLICPKNNQRWNNCGTFCPLKCSQLEPIPCTRQCVIGCECKPGTVLREDNQCVEPKDCNKILN
PpSPI49	PPU14690-RA	85	MSKFVVLCLLVLCISASLISVDARSPNRPLCPKRNQAWTDCGSACPPRCNQPLSQICTLQCIIGCQCKPGYLLNKNGECVKPEDC
PpSPI50	PPU14738-RA	83	MFTKIFAFCLMLFVSASLVSMNQAADHHDCPANQTYRDCNFICPPRCHLPITCLIVGVCAKAGCACSDDYMLKGDKCVLPKDC
PpSPI51	PPU01181-RA	922	MKLRKFSQLAALLLLATAAVNGSTHCEQKWDDIKAPHTVAETYRIKLLKINTTDEYRSFMPNTKYQVMIKNDIEDVQFIRFYITVENENKSLPHGLLELYDDELSEFTQDCPDAVVQVSQVVKDEISVYWTSPAEGNGCVIFRASVMESPSIWFMDGTLEQKFCQDPKASFDDPGPVLPECCACDEAKYELAFEGLWSRYTHPKNFPSKP

			WNARFSDVVGASHTSEYRFWEYNGYASEGLKQVAENGVTRVLESELKNQSQHIRTIIKARGINFPNITSKT<br>FAVFRVDQMHHLMSLVSMIDPSPDWFVGVSGLELCLSNCSWIEHKELNLYPIDAGTDDGITYESVDAETE<br>PRDVIRRITTTWPNDDRSPFYDDTAIDMNPLARLYLKRQRIYEKNCERSPSSEDSEGLGTTVDRPITRKACR<br>VTNWGPWESCSVTCGRGVKLRQRKYKNEKAAAKHNCDSLLTDRVVCYAPDNFVCPPDEVDEKKCPLSQ<br>WSEWTTCSKSCGPESRTRERNFRPKRKRKECRMQYPHIELQQTLDCENPACDGEETTTDGDSSTTGVAGD<br>EEAVSETTTESITTTISSDEESDNDAAENRRYLAKIWPRPRRKKCPESRYYQWSFWSPCSVSCGSGTKSRSR<br>QIKPDYSPNAADYECQYESAICEAETKSCQITPELMQVICSQPVEPGHCPDEHNDNSLRYYYDKITAKCLL<br>FHYTGCNGNMNNFRTLQECQETCSTFQDPDSLRSYRLKLSSVVTYNVPLTDPSNAKIKRAKSDENTTEKK<br>KKKKHKGETKSNDRVDCRMTEWSDWSPCYSCKGYRTMSRDVLTHPRNGGKSCPTKTMRRQKCHKVLP<br>DCDVQNDREALVFDSIIEEQVSVHCRVSPWGPWSSCATSCGLSQRRRTRKIAVEPRGLFGKSCPALAQIET<br>CRLPACPNAT
PpSPI52	PPU14032-RA	2035	MTSRGKWILGILTIAILLKLESCQATLSEEASDDVIVTPQTSTITSLFVGQQQQHATTAPVTTTTRRDLPDRC<br>FVKTEHGPCKNYVHKWSFNHTEGKCHTFVYGGCLGNDNRFNTEEECMHHCVGGPEHTLPPYMVTKGSV<br>FVTTSATVTSTTSSTTTTTPIPTFTPTKPTRPPVPKHKRGRELTFMESGYEKTFMFAQSNTFIQLDGPGIKTF<br>QLRLCREISFKFRTKLPHGLLVYHSVKDRPDRLDPYALYVIVEKGQLKVVHVFGKNSTSLTVGEGLNRDE<br>WHSVLVRIDVHGAKLIASVDGKQAETTVEVLEHIVNYGVSEELASVVLIGGLSSEERLHGVKYIIESFVGCI<br>MDMVLSSGKSASDLLPIRPLIATKHENVKEGCIDKCKTRENLCFLGSQCVNHYNSLTCDCFGTKYEGERC<br>DVYTATILTLRGSSYVSFRVYDWKDRVHSSVNRISLAFKTKWDDSALFYASGEIDGTAHYIAASILNGTLI<br>VELDFGHDSKIHTTFSHHITMNYWNNLTIFHNVSMVFISLNEEVKMLEVPGENYNMIIDPEIYIGGGPELNK<br>KQGLASTNNFAGSLKYVFFNDKSIIYELKRSNPMVHYIGVLEPEYYEDDVEVIPITYPFAGSHIWWPVEST<br>DSLKLNFDFKSFQPIAVVASGDVKSESGPGYWELRVVNDEIRFHLVPVLGENMTVTAAVKFPYNTSWHA<br>VELNYTREELSILVDYKNKQSKLLQMNLELGDRVIIGSGKGNAGLVGCMREIVVNDQRVEPRYIINTERV<br>VGEVALDNCQFVDPCKRPNTCEHGGKCSVKEDRITCDCTDTGYIGKNCHFAQYRKTCEELALLGYTKDD<br>VYRIDIDGNGRFPPALVKCEFQAIEDSTKTIVEHNLPSQVDVRSITESDFSFSISYVQFSAEMLQELISHSLYC<br>SQYVKYDCYKAPLELHSATWFLSSKGTPVDYIGNVNRGSCPCGVNRTCVTSNLSCNCDVSAGKWLSDEG<br>YYETPESLGITEMVFLQQRDLEEDAQGRITLGPLECVETNTQKYVVTFTTSQSYIEVPGWRKGDIAFSFRTT

			GERAILLYQPPIRSNYPSFMVALTSDYRLTFNFTLNTGTIRELEVKSRRKLNNGEWQKIWIDYNDYHVRFMINTDFQMVDLLPEEEFGPFEGSMFIGGATAEHLKTSSVRQGLIGCFRGLVVNGEILDIHSYMSVHLSEIIKDCKPSCQPNKCQNNARCVELWSNFTCVCENRWAHLGTYCETNINNKALTFTSPGAFLKKNYFASSEEEEEKTLLKSMLVKNILINLRTYDMHSLILYANDHLNNFAHLYISNGTNIVYLFNAGNEIKNITVEYPAANTGISVQIAIVRTENTTTLHVNENNVTLDAVPILLDSYSNKPWINPEKEILAPQRPPAPPTDYFQVNLGGFDPDNLLRVGAEGNLIQGYIGCLRGLMIGQYLVDLPNLASEANHEGSKGVLPNCHMKCDAVPCKNQGTCTEDFGKQEASCNCELTSYFGEYCADEKGADFSGESVLQREFDLNGEVNRIKIQLAFSTIDRQRTSALLLLQTENKRSYYLLVALTSEGQLIFEEDREGSAYGVRLNDRTFSNGARHSIYYVRDNNTATLLIDREQVQLMPIPALNSGDDEDDEGDDDDDGPGATEIQLGGLNTTDKRFRAYKGYTGCLSNVVVSINDGVGMKPLEEYMLFTKKGSETVRVTAPAGVRSAQCAVFHTQPRGLEPPKNDSVNRDRSWGEDPPKRVLYKSHFSDATQEEQGAGTYIFIALVCLFVAAVIGCSYEVWRSARKDRKRRKQRSRSGSSTSGSQRWPGYGADSGTTTPLSPGTKSVGFKTVVVDAEEKRPNGTHKTSGTPNAKDYKPLANSEPKAEPNALNDKRSIKDDEAEKKELLGVNTGLVSKPAKPNPFSMEDLREEPELEEEEQNEAVHEEDEEDEESETEPELTNTEISIEANGKPQVLTRPSEPGSRKNSTSQIPATWSNATAADQAKKLKNISLAPIFLSPDQRTFANPISYLGGPRLPASRSRSSIESILSLD
PpSPI53	PPU14848-RA	790	MTRSWHFKREEAMLRLLIAAWFVATAQAGCPLSPTSDQTSAKRLPGDGGYRILISGDYDKYIPNAVYTISLQGPHNYESRSQEFKRFTLSVDSQHAPFNPAARVGFFQIFPDSLTEFNEDCVNTVSEVSDYPKSEVQVMWRAPASGSGCVIFTAMVMEEPNRWFAEDGQLSRTFCEMSAKETESLDELRCCACDEAKYKIVMEGIWSNATHPKNFPDSAWLTHFSDVVGASHETNFSFWGRDHIATDGFRQLAEWGSASGLEAELRTKAKYLRTYIKMPGLWYPHVNSNTSANFKVDRKHPLLSVASMLGPSPDWVVGVSKLNLCQRDCTWIKGMTIDLYPWDAGTDNGISYMSPNSETRPREFMKPITTMYPEDPRSPFYDPSGKPMLPLARLYIDREEIKKKSCDERDLEDQVAEFNVQENTSDTERPECMTTEYSEWTACSVTCGKGLRMRTRAYRIPEKAAMMGCSRQLVSKEMCLAPAGECSGGSSPAAADDDNPFPGLDNAECETSDWSDWSECSSSCGVGFKTRWRRLRDSSKRKKCFHVSLMEKEKCMQPPCAPGTEEKQDEVCKVTDWSDWSPCSATCGTGVKTRTRLLLVLPELQTECARKIQLRQQRPCLDQADCTFDVATAKAICMEKPEKGPCRGYFERWVFNPEKRTCVPFIYGGCRGNRNNFRTSAECSKTCDVVRETLGGGQPPSQSLDPLPPRHSTNDRLIASAASDQPARSLAPVDCVVSDWSPWTACSVTCGTGRVTSSRYITRQPTNGGKPCPKRLQRRARCQLAPCDDY

PpSPI54	PPU12795-RA	2827	MLYGHFGPILLLPIDLYHHIKLRHDRHRRQQGESYAPPSHVLDSEEPERGSWGPWSHPSACSRTCGGGVA<br>SQTRECLDRDDYNNDRCTGAKKRFFSCNIQPCPGEQKDFRAEQCAEFNNKPFEGVYYDWIPYTGGHNKC<br>ELNCMPRGERFFYRHRESVIDGTLCNIEKNDVCVEGKCMPVGCDMMLGSSAQEDACRECGGDGSDCNT<br>VKGLFDQDDLQAGYVDILLIPEGATNVVVREIKPSNNYLAIRNTTGHYYLNGNWRIDFPRSLRFAGTIFHY<br>SRDPQGFSAPDTITCLGPTSEAIYVVLLYQDHNVGVEYQYSMPKKFSQQSDPDSYTWVADEFSTCSTSCG<br>GGYQSRRVSCVKRRTNEEADEKLCDPMLEPADTQACGQDPCPPQWVASDWSNCSKHCGEGGEQTREIK<br>CEQIVSGGVPTIVDENQCIERLGPKNVTKQQCNKDVECPQWHLGPWKPCDHLCGEGKQTRKVTCYKKNE<br>QGKVQVLEDSACEGEVPEKEKACELRPCEGVDWVVSPWSGCSDKCGLTQETRTVQCATQGGKVYPENM<br>CDSEKKPESTKECETSQNCEYQWIKTQWSKCSAQCGTGIQTRKVFCATFEDEDTMKKVPDSKCEAEEKF<br>NATRECTAKSEECKGEWFAGPWSKCSKPCGGGESTRKVICLKDNKTVSSEQCDYQSIMEETEACNTKAC<br>EEDEVLPVEPSKVTALVPDEDEECEDEEDEDFVTIDSRFNTDESEKSSDMTEASPLSSPLASDSTDMFSSTL<br>EDRMYSDGISRGDVSAPTDSGSGSTSSDDDEFSVPILTTVEGSGTSPDEFDLLTTISGSSDTAATESSDDITE<br>GSGTAESAVTESGFSASGETVAANSGATEVSTDGTTSGVTDQSSDQTTEQSTEQVTEQSTDNASAASSTEE<br>STGDTEVTSDSSAMTTDSSTDSSVSSSDTTDSESTEPTDTTPTSDTESTEATSDSSASTDSSTMASAISETTFE<br>SSTEPNTESSGASSDAGVSTESSGSETTEGSVESSGSTEAVTGESGETTVAGTDVTEASSDTSESSTLVSGET<br>QTVESVTSDTTESGATETGATETGATETGATETGATESSDVTESGATQESTVEGTTESGMTTESGETEATS<br>EYSTIGATDETTESGVSTATEETDLTGQSEIMDLFTTPSAVDKAITIEHRIKKCKPKKKKTCKTTEFGCCYD<br>GVTAAQGPFGKGCPTPHTCQETNYGCCPDGVSPAAGPKFQDCPDQHCGETLFGCCEDGVTPAEGNDFEG<br>CKKPCNQTEFGCCPDKETPASGKNNLGCCNTTEFGCCPDGIKPASGKDGEGCEEEAETDTPIETTTLVPGD<br>CANSTYGCCPDGQKAANGTNFEGCGVIDTKNCTASYFGCCPDNVTAALGPNNTGCHMPCDNTTYGCCD<br>DKETPAHGPNKEGCCLSSQYKCCPDNILPARGPNFYGCGCQYSKFRCCPDNTTAARGPNNEGCGCQYTP<br>HGCCPNRFTPATGPNYSGCPCYTYQFGCCPDGVTTAKGPHGQGCGCENTEFKCCSDGRTPAKGPNFAGC<br>TCDASKYGCCPDGIEEAQGENFEGCLKVPSIPSAACALPRDRGTCREFTVKWFYDTEYGGCSRFWYGGCE<br>GNDNRFKTQEECKAICVEPKGKDVCYLPKITGPCEGYHPTWYYDTDRKQCGQFIYSGCLGNGNRFKSRE<br>ECEERCAEPADADPCTLPQEAGPCEGNFTRWYFNKESQNCEIFKYGGCKGNHNNYPSELACRQQCLQPG<br>RSRVMQDVCALDKEPGPCPGSLLRWYYDANRETCRKFIFGGCKGNGNKFRTRAACEQRCRVRDSCSLPR

			AEGNCTDKFSRWYFDQQENRCMPFYYTGCGGNKNNFGSRDACESDCPPKIEQDICLLPALLGECHNYTQ<br>RWYFDSYEQRCRQFYYGGCGGNDNNFHTEHDCQNRCEGPVNTPTPPTIEFRSDFCFLPDERGPCQNYQN<br>KWFYDSREGICKQFVYGGCSSNGNNFNSREECEYRCGEVQDPCTMPVLVGPCNGSVPQFYYDRRADAC<br>YQFDYSGCQGNKNRFQDVSSCEHRCRKRPAIPATPASTRLPENNTPLSPGCLEPVEAGPCDGEITAYFYDK<br>DAGKCQAFIYGGCEGNANRYETEEQCERLCGQFREQDICHLPVDQGPCRGSFPKFYYDQASRICREFTYG<br>GCDGNANRFSSRNECESVCIHHEEPAQSGNKTALSDLTICKEPVDIGSCSVGNYKRFYYDDEYQTCRAFIY<br>TGCGGNRNNFKTIDSCLKVCRQINAIPDDGQDTKDPCAEATEHCHQLHCPYGQEDYVDSQNCQRCRCNE<br>PCQSVQCPEGTKCAVTLVGSSDGTTYRGVCNPMTKPGKCPVVSNSTRCEIECYTDADCSGEQKCCRSGPC<br>TSCLNPATEEVITTPPPYVPDEQEAIPPGAVPAKIEQPESPNVSAQEGGYVTMRCVVIGTPAPIIVWRKDAKI<br>IGSNENRRRLLNDGSLQIINLYPYDKGLYVCTADNGIGSPVRIEYQLDVLEPTDTSPGIIDEPNRAITVTLNS<br>PTVLHCYAVGWPRPTVTWWRNDSMLPLSSEHYEQESDNTLRIRSVTLSNLGVYTCHAFNGIGKPAEWSTI<br>LQAIGPVANIRPDQEQYRKYLVQAPQRPERPSYPYRPNRTQIHENQTYAPIYTTKRYNIPGVVPINITTPTPE<br>VDYASPVRVNVSASQVQFPEGGPIKLYCNVTGSPTPRVTWYKNDEPIQADERIRISESNELTISPANSNDTG<br>TYRCEGVNQHSTSSESVDIRVAGIYIDPLCQDKVHLANCSLIVAARYCQHEYYAKFCCRSCTEAGQLPSRV<br>VPPYADNQRAKRSLFSFL
PpSPI55	PPU07478-RA	1887	MRRDLSILLCLAALSCAAGLNRNRGYIFTAPKRLIAGETENVCLSLHNLEPPVHVSVDLLATSSAAGAVTP<br>ASAGGVLVEDDILGSVKNTLKSGVETCLELRVTSSTKYSAAKLRLRLKFDKHPEYVVDTEKKVFIEHDAL<br>VTFVETDKAIYKPGQDVDIRILTLRHDLKPWIKAIPKVWIENPSEVRVAQWTNVTTENGMAQLSFPLSTEP<br>SSGIWRIKVEKNRPQLVHTSTFEVRKYVLPRFQVTIGAPSYILADAQNATWKVCVRYSYGEPVKGKLLLS<br>LRPQTPIWKRKQTVADINYEETLDAKSDGCMNYTVSAQVLGLPHWKVAPNNVVLLANFTEAKSGVVET<br>ATSRTPVMHQPLKLEFSPHTLKYFKPGLPYHGKLRVLRADASSPAPNEKIQVCLRIRRKDEWQRSVVECR<br>NFTSSNSDGFLDFIVPPQNKNIVLLSFVATAVNYPTKYYSPDKRWRVFVDQPSAYIDVEPWYSPSSSYLSV<br>TRGSQPLVCGEKYSFNVMYTSGTSSTVNATNLSPDDTEPISFHYSINSKGDLLVFGHVKYKPRKDTLLDYS<br>EFQHVLGAGVGSKPNPSVHRFPLSVKITASMAPVSELLLYYVRPDGEVVTASHSIEVGHCFENKVKTAWQ<br>QEKQNPGSLAKFHVEAAPLSLCGISAVDKSTRFLTQSQPVSSEGGSASSPGSNLLEPEATFARLKPFHLPPE<br>TMPLQSTWAHCDKSQAAEDSNSEEGPMEEIDHLPKPAARNKRHSVTYNVAANYVDAIQAFDDFGTIVMS

	DLILESRPCPPWRSSFGRVPLGPSSDEIDEPDRMLKAVKAMPLAFQFGAPGIPGPEASPMGQDVNYVDPTS<br>MESQTATIVRSYFPETWIWELVPTGKDGRATIERQLPDSITDWIGNTVCISTKSGLGIGNPVQITSFQPFFLD<br>YSLPYSVKRGEQLRLKVSLFNYMQHSLPVLIKLLDHEGLDLGFNSSSEASYCLGPRDSIVHGFPLLPRELGE<br>INVTVAAEVDKERAVACGAPTPLPDNRDEIIKPVLVKAEGFPVEISRSSFLCPRDFSDDTSLVWELELPRPE<br>DEQVVEGSASAHVSLIGDVLGPALENLEQLVRLPMGCGEQNMILFVPNIHAIAYLDAINRQTGSEMRARAI<br>KNMQKGYQRELNYRHPDGSYSAFGAAADEAGSGSMWLTAFVVKSFAQARSIIQIDERDLKLSVKWIVRR<br>QLENGCFPVVGQVFHKDMKGGLREEDGSSSALTAYVLIALLESGVPLSAALVNNALYCLEKASASDHFA<br>DNPYTGALTTYALALLEHPRANESLRSLMGRASRQKDLLWWEDKSRPGSLALSIEMTSYGLLSLIKLGGE<br>NNTMEALRVVRWLSKKRNAEGGFSSTQDTVLGLEALTKYALKMANASATELSVLLTANDMEKLFKIND<br>ENRMLLNRVELPTLPTTLEVFAEGEGCLLVQSSLRYHKAKASGSEAFDLSASTVSISTADQTPNDGCSIQR<br>LTVCTRYKLPDEESNMAVLEIAMVSGFRPDRASLHDLLDEHATGVKRFEENDDTVAIYFDKLTAQKTCIS<br>FQAIRENVVDNAEPANIKLYDYYQQELTVSTNYKFMDVCEKNKTEKAVVNHDANNPAPRKMQQLDNRL<br>SVNQLPEKVDGIFEKDMTREKKVDPSIFKQRSSGVTIPATISIDLDEGSGIEPDGQLFSPSPILPNIDQDVVDE<br>GFSPAFIDVDHELETPNGIEGPIPFYVEAPVSTTTKESVFAQTPLNSKPSLNFHNSTKLKTEETKISRSLASCP<br>RCEEGIPSDIKELYCSSESAVKVAIRRSRKARLLLDLSSSNASTRLRSTIELNIKPECSCAPLDKPGIMALLLS<br>SKDGFNFAKSSDKSKALLDRSNSMFALTSVVGTPHEIQEAQLSCSRNP

PpSPI56	PPU08491-RA	1702	MPNWSYFVRDYETVCSRNMRRGVTAALALLALVGISRAQFPPEQTEAPRLPWSRFDDGREQNFRDRDRDQDRVEFRDRDQNRDNSLGNSGSTRDWDSINRDTSSRDRDSNFGFGYRATSQQNENVIIKEATYFIVASRMVRPGQVYRVAVNILRSPLPMMVRASIQRNGVEIAADFQEVREGIPEKLMMRMPPTSVHGDYRLRVEGNYNSLTGGQAFLNETTLTFTQRSMTIFIQCDKPVYMQGQTIRFRTIPINTELKAFDDTVEVHMLDPYRRIMRRWLSKQSNLGTVSLSYQLSDQPVFGEWIIKVIAQGQVEEKAIFVEEYYQTRFEVNVTMPAFFFDNEPYIHGIIQANYTSGAPVHGNLTLRASFKPINKYSQTTGAMENIPDRYFNFNEYYPAWFNPPDLFRETVPVLRFFNGTYRFQYPMSELLPYLPNTSEGMEVTVTATVGERFLEEIIVGYSTARIYNSSIKIHFFGGSPQVFKPGMPFDLNLVASFHDGSPLRAAQLRGAQLEVRGDIEMRSGRRSLENQIVKVSPENAAVWSLQIDLRKQFESSQGPQHTQQLLNEIISMRFYADLTDGEGHHGHAELLMLAHESPNQKHIKVWTSTEKAKVGEYLVLHVQSNFYIESFNYLVMSKGTILLTSDERMQQSIKTFAVPLSPEMAPVATVLVYHVGKYGDVIADSLTLPVNGISRNNFTVFINNKKARTGEQVEVAVYGEAGAYVALSGIDRSFYAMQAGNELTYANVIEKMSNYGEQTNGTHAHTWLFHEGDPDLVVNFPSSTFGIDVNRTFEYIGLVAFTDAVIYRRPEHCNRTLGYLECLTGRCYRVEQQCDGIMQCDDGTDEARCPVGNATDIAHFRKWRFNRIQRQYENVWMWRDINIGPHGRFIFNIDVPKTPVHWMITAFGMSPTNGFGMLPSALEYIAVLPFYINVEMPMHCKQGEQVGIRVSIFNYMRYNIEATVVLADSRDYKFVHVEDNGIVESYSPRTSFGEHQFFLWIPAQDASIVYIPIVPVRLGDIKVHISATTLIGKDSATRTLHVEADGLPQYRHQSILLDLSNRAYVFQYMHVNITETPIIPYEEDRYYIFGSNKATISVVGDVVGPIFPTMPVNATSLIGLPMDCGEQTMFSFAANMYTTLHMRLINQRNLTQEKQSFYYMNIGYQRQLSFMNPDGSFSLFRSDWNVSHPSVWLTAYCVRIFEEASFYEWENHLYIDPKVIAQAVSWLLDHQTYEGSFYEVTWLPDRKINSTLHYDGDENHYPYKSSRNISLTAHVLITLETVKDLSDGLGARVALAAANAVRYLEQNLQLLELGGRPYEIAIVAYALLQAKASTAEQAFNLLRRYRREEGGLVYWGREMVPQPPYKTENQKPFLLPRLPYKYDSENIETTAYALLVHVARQEVEVEPIVKWLNSQRLTDGGWASTQDTAWAMKALMEYTVRSRIRDVSQLSVTIEATALPGQTNILHVNNKNLARLQTLHIPEAWGTVKVQAKGAGYAILQMHVQYNVDIKRFQTKPPVPAFDLVTRANFYGRNQSHITYLSCQRWINVNESERSGMAVLDVTIPTGYIIQQQNLDRYILSRQVRNLQRARFQPSKVLFYFEYLDQEETCVNFTIERWYPVANMSRYLPIRVYDYYSPERFNETMFDSLPTYVLNICEVCGSSQCPYCPIYNAATLLSPPGLLITMMSLFFIFRRRNLEVG

PpSPI57	PPU09870-RA	1450	MDWSPWPLLLLFCGLASGQSYYSIVAPRVVRPNSEYHVAVSIVGVSEPTTTFVELSGQLDSGESFAVSENII<br>VEPYATRVLSLEIGDTGPGRYRLLARGISGYEFVNSTELDYAHKSYSVFVQTDRSVYKPGSKIQFRCIVLDS<br>RLRPTANRQLEIYITDGQGNRIKQWERPRLHQGIFNGELELSQSPVLGDWEIVAVIGGQTFKKGIQVAEYV<br>LPKFEVTIDSPPHATFKEGKITVLVHAKYTYGKPVKGEATITAFPDIYSGVLQPIYSPPIRKTVNIDGKTTVD<br>FDIANDLKVKDDDYKRPVVIEVAVEEAVTGRRQNNSMQITLHKHKYTMELLRTAEYYKPGLKYTAFLKV<br>TYHDGSPVVDNTNPVHISYGYTYDSEDLHNITRMLDKNGMIELDFYPPLSVPDKIFRPLRIEAQYLNLHEW<br>FPSTNPATSRSESYIQAMLRTDKPKVNEYVEIEVNSTHPLKYLSYQVLGRGDVLNAASIQISDRYTASFRFL<br>ATYVMAPIAHVVVHYVREDGELVADSLDVELEGTLQNFVDIKPVSDEVGPGDNIDLTITAKPNSYVGLLG<br>VDQRSLLLKSGNDITYEQVRKELMSYDVNDAAFYDQEDYAHSWIRPGSASTDEVFRKTGTVVLTNGYIH<br>ENPQLIYYRNSMDSVMFSAAVSESAATYSPEALKVRKNFPETWIWETVDMRYEGKTVIRRTVPDTITSWV<br>LTGFSVDPAFGLGLIEAPRKLRVFKPFFLSMNLPYSVIRGEIVAIPIVVFNYMSKDLNVEVVLENNGDFEFA<br>EVSNEVHDNTKRLELYRTKKIFVKANSAESVAFMVIPTKLNHITIKAKATSVMAGDSVEYPLLVKAEGET<br>QYRNKVVFVDLRDTDSMKTNVTVDIPKHFVTDSEYVEVSAVGDILGPSIPNLSKLIKMPFGCGEQNMLNF<br>VPNIVILDYLKNTNQLSPAIESKSIRYLETGYQQELTYRHTDGSFSAFGKADPSGSTWLTAFVAKSFKQAE<br>KYITVEEKIIADALKWLAEKQAANGSFPEVGTVSHRDMQGGAAKGLALTAYVLSAFLEVENIEGRYRNVI<br>YKGVDYVVRNMQGIDDNYALSICTYVLSLARNAYEDEAFRLLDSKATTKDEQKWWSKPIPEDDKKNPW<br>FSLSRTVDVEMTSYALLAYLRRNQLSDATAIMKWLVKQRNAEGGFASTQDTVVGLYALAKLGEKLRTN<br>VYDVQVRITTDVGESKEININSRNFMIVQKHLLLGRTRAINITATGTGFALVQVASRYNLNVTGAFPLFTL<br>DPQVDKISTNDHLQLSICSGFIPTKEANESNMAVMEVSFPSGFTVDQDALPSLELSQNVKRVETKNGDTM<br>VVLYFDKMVHDKPYCPTVSAYRTHKVAKQKPVPVSIYDYYDSSRRARVFYEPKMTTLCDICEDEQCGDV<br>CSIKAGKREDGTLPSVSSSSTTLVDIALILACASYVALNSRR

Supplementary Table S3: Predictions of SPIs in *Nasonia vitripennis*.

SPI domain	Number of SPI domain	Gene ID	Size(aa)	Signal P/TM[a]	pI/Mw
serpin	1	Nasvi2EG019672	443	SP (1-24)	6.18 / 49310.81
serpin	1	Nasvi2EG007825	405	/	6.66 / 45827.52
serpin	1	Nasvi2EG007817	413	/	5.49 / 46047.64
serpin	1	Nasvi2EG004051	410	/	5.70 / 46955.77
serpin	1	Nasvi2EG001465	640	/	5.94 / 69517.98
serpin	1	Nasvi2EG001417	625	SP (1-18)	7.03 / 70485.97
serpin	2	Nasvi2EG036959	1004	SP (1-19) TM (5-22)(86-108)(110-132)	7.97 / 110674.82
serpin	1	Nasvi2EG007826	382	/	5.21 / 43879.26
serpin	1	Nasvi2EG007190	158	/	9.30 / 17941.03
Kazal	4	Nasvi2EG013640	521	SP (1-20)	5.85 / 57937.00
Kazal	8	Nasvi2EG003124	497	SP (1-22)	8.18 / 54774.62
Kazal	4	Nasvi2EG013643	289	SP (1-18)	6.77 / 33067.47
Kazal	2	Nasvi2EG013642	206	/	5.76 / 22636.02
Kazal	2	Nasvi2EG018573	207	SP (1-19)	9.06 / 22411.95
Kazal	1	Nasvi2EG017583	87	SP (1-25)	6.68 / 9984.57
Kazal	1	Nasvi2EG003930	89	SP (1-22)	3.90 / 9761.87
Kazal	1	Nasvi2EG013639	120	TM (39-58)	8.75 / 13079.22
Kazal	1	Nasvi2EG020608	85	SP (1-23)	5.63 / 9288.74
Kazal	1	Nasvi2EG020606	82	SP (1-23)	6.53 / 8954.28

Kazal	1	Nasvi2EG013638	80	SP (1-23)	6.01 / 8545.88
Kazal	1	Nasvi2EG020609	77	SP (1-23)	6.24 / 8343.69
Kazal	1	Nasvi2EG013644	78	SP (1-22) TM (35-57)	7.54 / 8180.49
Kazal	1	Nasvi2EG020607	76	SP (1-23)	8.68 / 8238.69
Kazal	1	Nasvi2EG013736	113	TM (52-69)	6.55 / 12854.96
Kazal	1	Nasvi2EG012972	172	/	8.70 / 19217.09
Kazal	1	Nasvi2EG009506	110	/	5.87 / 12497.66
Pacifastin	5	Nasvi2EG009665	296	SP (1-17)	7.21 / 32722.23
Pacifastin	7	Nasvi2EG009663	340	SP (1-16)	7.70 / 36902.97
Pacifastin	10	Nasvi2EG009667	564	SP (1-16)	5.32 / 62337.83
Pacifastin	2	Nasvi2EG013008	315	SP (1-21)	6.23 / 35214.48
Pacifastin	4	Nasvi2EG009664	240	SP (1-17)	7.72 / 26119.74
Pacifastin	1	Nasvi2EG015696	267	/	4.58 / 29117.10
Pacifastin	2	Nasvi2EG013010	255	SP (1-16) TM (164-186)	8.62 / 28818.31
Pacifastin	1	Nasvi2EG007350	414	SP (1-24)	8.33 / 46018.54
Pacifastin	2	Nasvi2EG012894	257	SP (1-19)	5.96 / 28235.91
Pacifastin	1	Nasvi2EG000669	133	/	5.01 / 14860.63
Pacifastin	1	Nasvi2EG000668	73	SP (1-24)	4.79 / 7874.01
Pacifastin	1	Nasvi2EG000667	95	TM (7-26)	8.21 / 10768.49
Pacifastin	1	Nasvi2EG009459	74	SP (1-26)	8.53 / 8089.51
TIL	1	Nasvi2EG000352	84	SP (1-23)	5.51 / 9093.71
TIL	1	Nasvi2EG000351	84	SP (1-23)	9.03 / 9169.98

TIL	1	Nasvi2EG000380	83	SP (1-23)	7.95 / 9162.79
TIL	1	Nasvi2EG000378	83	SP (1-21)	8.55 / 9017.61
TIL	1	Nasvi2EG000379	83	SP (1-23)	5.65 / 9136.66
TIL	1	Nasvi2EG000354	84	SP (1-23)	6.03 / 9087.65
TIL	1	Nasvi2EG000359	83	SP (1-25)	8.54 / 8713.39
TIL	1	Nasvi2EG000280	83	SP (1-24)	5.78 / 8927.45
Kunitze	1	Nasvi2EG009175	934	SP (1-23)	5.99 / 106581.75
Kunitze	1	Nasvi2EG017828	2075	SP (1-25) TM (1772-1794)	5.59 / 231846.76
Kunitze	1	Nasvi2EG009433	779	SP (1-16)	6.18 / 87215.49
Kunitze/WAP	11/1	Nasvi2EG011720	2549	/	4.59 / 278580.09
A2M	1	Nasvi2EG001033	1719	SP (1-20) TM (1688-1710)	/
A2M	1	Nasvi2EG002345	1534	TM (1507-1529)	6.45 / 172352.24
A2M	1	Nasvi2EG014366	1888	SP (1-19)	5.90 / 208217.69

a SP, signal peptide; TM, transmembrane region; /, neither signal peptide nor transmembrane region detected or incomplete gene (missing some exons or 5' terminus and 3' terminus).

Supplementary Table S4: FPKM values of the *Pteromalus puparum* SPI genes at different development stages and tissues obtained from the RNA-seq data.

Gene Name	Gene ID	Embryo	Larva	Pupa-FM	Pupa-M	Female	Male	venom	Carcass	Ovary
PpSPI1	PPU03966-RA	7.812	3069.610	623.890	451.520	782.313	584.295	190.996	100.096	89.845
PpSPI2	PPU05464-RA	20.003	7.905	20.665	20.698	12.237	2.717	19.495	12.992	20.015
PpSPI3	PPU06944-RA	172.981	113.338	113.881	103.897	154.404	31.567	145.889	91.886	133.447
PpSPI4	PPU06883-RA	10.042	32.326	24.459	29.646	68.784	73.246	23.435	11.492	34.368
PpSPI5	PPU01309-RA	42.947	162.440	271.385	127.749	75.540	81.982	16.607	42.132	23.893
PpSPI6	PPU06845-RA	0.017	0.018	0.287	0.083	0.221	1.151	0.038	0.039	0.082
PpSPI7	PPU03962-RA	34.977	200.415	93.128	44.041	123.538	65.974	26.956	28.870	39.129
PpSPI8	PPU05464-RA	20.003	7.905	20.665	20.698	12.237	2.717	19.495	12.992	20.015
PpSPI9	PPU06096-RA	4.108	0.489	1.128	1.795	2.604	0.136	0.353	9.736	4.634
PpSPI10	PPU03963-RA	0.000	4.079	1.445	0.832	1.811	0.616	2.019	0.551	0.296
PpSPI11	PPU00458-RA	0.136	2.789	7.395	10.891	2.593	4.443	0.083	0.757	0.387
PpSPI12	PPU03796-RA	0.000	82.969	20.314	19.886	178.996	67.200	6.944	167.864	3.722
PpSPI13	PPU05137-RA	0.235	2.717	38.572	34.443	9.904	8.484	0.926	0.540	0.941
PpSPI14	PPU05579-RA	0.000	0.000	0.000	0.000	0.000	0.000	0.000	0.000	0.000
PpSPI15	PPU07660-RA	0.000	0.000	2.655	4.237	0.000	0.000	0.000	0.000	0.000
PpSPI16	PPU12052-RA	0.000	1.255	0.000	0.000	1.109	0.000	16.566	3.259	0.000
PpSPI17	PPU12053-RA	171.974	498.295	93.207	63.903	61.954	33.207	33.904	53.147	112.628
PpSPI18	PPU12054-RA	0.000	0.234	0.000	0.235	111.283	0.000	6193.810	5.458	1.490
PpSPI19	PPU12067-RA	97.613	584.472	594.859	711.144	1004.991	1319.960	313.635	379.436	61.302
PpSPI20	PPU12265-RA	0.000	491.961	20.019	33.371	43.327	6.797	2448.760	8.148	3.959
PpSPI21	PPU12266-RA	0.000	18.521	0.000	0.000	0.000	0.000	0.000	0.000	0.000

PpSPI22	PPU12267-RA	0.507	446.781	0.000	0.000	0.554	0.971	3.012	1.630	0.000
PpSPI23	PPU12268-RA	0.000	74.500	1.043	0.000	1.605	5.911	19.132	1.367	0.000
PpSPI24	PPU12269-RA	0.000	1630.940	12.600	26.015	2152.329	802.513	23469.200	426.631	11.911
PpSPI25	PPU13579-RA	0.557	774.996	57.839	79.062	401.536	935.118	101.026	459.732	2.180
PpSPI26	PPU13580-RA	3.472	19261.200	111.270	270.484	1074.619	1476.790	292.347	988.992	0.492
PpSPI27	PPU13581-RA	0.391	1.405	1856.210	1434.190	0.812	1.827	0.271	0.828	0.141
PpSPI28	PPU13582-RA	0.000	0.258	83.045	50.857	0.079	0.215	0.000	0.459	0.049
PpSPI29	PPU13583-RA	27.827	17.606	10.584	8.260	735.351	208.674	1150.790	280.388	19.338
PpSPI30	PPU14014-RA	0.000	0.000	0.000	0.000	431.293	0.000	30894.800	40.317	2.655
PpSPI31	PPU14466-RA	0.618	2.031	30.378	32.103	6.267	3.793	2.200	0.945	0.523
PpSPI32	PPU16162-RA	0.000	1.522	0.383	0.376	0.242	0.000	0.000	0.000	0.000
PpSPI33	PPU02466-RA	0.000	0.095	0.000	0.093	927.907	107.966	2170.600	7.719	1.219
PpSPI34	PPU03404-RA	0.094	204.183	0.238	0.494	14.325	2.949	473.841	59.253	1.142
PpSPI35	PPU07367-RA	0.000	171.285	3.962	1.265	0.199	0.415	0.000	0.184	0.000
PpSPI36	PPU07368-RA	0.023	266.107	76.970	7.518	57.725	39.244	1.446	5.743	0.355
PpSPI37	PPU07369-RA	0.000	323.636	46.642	8.973	132.269	76.208	3.128	4.742	0.423
PpSPI38	PPU07370-RA	0.044	324.837	1.609	0.696	9.703	8.303	0.000	0.152	0.150
PpSPI39	PPU07670-RA	0.841	1892.210	0.000	2.588	0.613	0.224	0.000	0.149	0.472
PpSPI40	PPU07672-RA	0.063	631.045	2.691	3.068	19.513	8.462	72.907	100.827	3.436
PpSPI41	PPU07674-RA	0.000	7.558	6.779	8.672	3.209	10.555	1.275	16.403	0.577
PpSPI42	PPU08700-RA	0.134	0.000	0.000	0.000	0.000	0.000	0.000	0.000	0.000
PpSPI43	PPU10736-RA	0.072	0.037	0.180	0.107	2.438	11.298	0.000	2.156	0.035
PpSPI44	PPU14859-RA	0.000	1126.400	1.737	1.691	7.373	60.221	0.000	3.339	1.014
PpSPI45	PPU09199-RA	0.047	24.472	0.095	0.328	0.079	0.204	0.639	2.504	0.046
PpSPI46	PPU14671-RA	0.000	1.191	0.000	1.656	971.527	296.411	76.702	1041.380	0.630
PpSPI47	PPU14688-RA	0.000	726.851	0.000	0.000	0.274	0.000	0.000	1.531	0.000

PpSPI48	PPU14689-RA	0.386	2451.570	0.901	6.619	332.833	51.792	115.456	72.009	0.000
PpSPI49	PPU14690-RA	0.000	3011.460	0.000	25.542	74.281	11.734	38.833	23.350	21.043
PpSPI50	PPU14738-RA	0.000	145.519	0.000	0.000	12.179	6.621	0.000	7.223	0.000
PpSPI51	PPU01181-RA	1.040	0.322	3.176	3.062	3.129	8.156	0.842	2.219	0.780
PpSPI52	PPU14032-RA	0.023	1.239	1.719	4.157	4.355	4.288	0.304	1.446	0.689
PpSPI53	PPU14848-RA	0.293	28.949	31.301	31.292	34.920	23.766	1.205	13.230	7.911
PpSPI54	PPU12795-RA	0.049	5.728	16.062	24.925	2.974	0.933	0.000	0.893	9.932
PpSPI55	PPU07478-RA	0.259	9.555	32.618	36.424	7.297	8.436	0.069	2.387	0.442
PpSPI56	PPU08491-RA	0.123	19.084	37.459	39.996	3.337	2.508	0.809	1.554	4.459
PpSPI57	PPU09870-RA	6.510	13.517	28.988	20.646	18.316	10.838	2.542	6.519	11.483

Supplementary Table S5: Differentially expressed *Pteromalus puparum* SPI genes in the venom gland.

Gene name	Gene ID	Signal P	Proteomics	Venom FPKM	Carcass FPKM	log2(Fold_change) normalized	z-score	p-value	q-value (Benjamini et al. 1995)	Signature(p-value < 0.001)
PpSPI18	PPU12054-RA	SP (1-20)	/	7638.45	6.764	9.964	73.710	0	0	TRUE
PpSPI20	PPU12265-RA	SP (1-23)	/	450.171	2.250	7.468	20.668	6.69E-95	4.01E-93	TRUE
PpSPI23	PPU12268-RA	SP (1-23)	/	19.132	1.367	3.630	4.027	0.0000564	0.00014454	TRUE
PpSPI24	PPU12269-RA	SP (1-23)	Y	23469.2	426.631	5.605	154.929	0	0	TRUE
PpSPI29	PPU13583-RA	SP (1-22)	/	1150.79	280.388	1.860	21.7592	5.65E-105	3.63E-103	TRUE
PpSPI30	PPU14014-RA	SP (1-19)	Y	30894.8	40.317	9.405	154.231	0	0	TRUE
PpSPI33	PPU02466-RA	SP (1-20)	Y	2170.6	7.719	7.959	44.425	0	0	TRUE
PpSPI34	PPU03404-RA	SP (1-19)	Y	326.045	43.438	2.731	14.619	2.14E-48	6.5E-47	TRUE

Supplementary Table S6: Primers used for qPCR on gene expression.

Gene Name	Gene ID		Sequence (5’-3’)
PpSPI4	PPU06883-RA	-F-	CCTTAGCCATGGTGATGCTT
		-R-	GATCTCGGAATGATGGGAGA
PpSPI5	PPU01309-RA	-F-	GAAATCGAGACCCAGGACAA
		-R-	TTCTCGAACGCGTAGAACCT
PpSPI18	PPU12054-1-RA	-F-	CAACCAGTTTGCGGTACAGA
		-R-	TCTGCGTCTGAGTCGACATT
PpSPI20	PPU12265-1-RA	-F-	GCTACTGCTGGCGATGATTG
		-R-	CGCCAGTTGTCGTAGGTCTG
PpSPI22	PPU12267-RA	-F-	CTTGACGCTTTGTGTACTCCTG
		-R-	GCTTCGCTTGTGTCTTCCTC
PpSPI23	PPU12268-RA	-F-	CTTCGTTGTCTTATGTGTATCCCT
		-R-	ACTGATACCAATACTCGTTCTCTG
PpSPI24	PPU12269-RA	-F-	GCCAAGTCTATGCTCATCGT
		-R-	TAACCTGGATGTTGGATCGT
PpPSI29	PPU13583-RA	-F-	GTTCTCATTGTCGTATTTGCGT
		-R-	TTTGGTAATAGTCGTATCCGTG
PpPSI30	PPU14014-RA	-F-	AACAAGCGCTTGATATCTGTG
		-R-	CTTGACAATAGTGAGTTCTGTTCC
PpSPI33	PPU02466-RA	-F-	CCAGTGATCCGGCTTGTACT
		-R-	CGGCGCAGTTACAGTCATTA
PPPSI34	PPU03404-1-RA	-F-	TGCCATCAGTGCATTTGTGA
		-R-	TCCCGGGTTGCATTTCTTTA
PPSPI37	PPU07369-RA	-F-	CTGGAACAAAGACGGCTCTC
		-R-	GTTCCATCCTCAGAGCAAGC
PPSPI38	PPU07370-RA	-F-	CACATCTTTCGCCTGCACTA
		-R-	TCGTGCAGGCTTTGTTACTG
PPSPI40	PPU07672-RA	-F-	GCACTCCTGGAGCTGTTTTC
		-R-	TGCCAATAATGTTGCAATCC
PPSPI43	PPU10736-RA	-F-	TCTCTACAACTACCACAAATGCC
		-R-	TACCTGTAAAGTCCAGATCCTC
PPSPI47	PPU14688-RA	-F-	GTACCCATTGCCCTCAGAAA
		-R-	CTTAAGGACGGTGCCAGGTA
PPSPI48	PPU14689-RA	-F-	ATGTCCAAGTCCATCATATTGTGTC
		-R-	TTAGTTCAAAATTTTGTTGCAGTCC
PPSPI49	PPU14690-RA	-F-	TGTGTCTCCTCGTTCTGTGC
		-R-	GTGGACAAGCAGAACCACAA
18s rRNA		-F-	CGAGCGATGAACCGACAG
		-R-	CGGGGAGGTAGTGACGAA
Actine 1	PPU09639-RA	-F-	CTCGCTTTATGCCTCTGGTC
		-R-	AAATCACGACCAGCCAAATC

Supplementary Figure S1: Sequence alignment of 10 *P. puparum* serpins and serpins from *M. sexta* Serpin-1K (MsSRPN1K) and human alpha-1 antitrypsin (HsA1AT). Conserved residues involving structural conformation of serpins are highlighted, based on the amino acid residues of HsA1AT. Residues corresponding to the shutter are highlighted in blue, residues corresponding to the breach in yellow, residues of the hinge in green, and residues of the gate in violet. Other conserved residues are highlighted in gray. Predicted helix and β-strand secondary structures of serpins are assigned based on the crystal structure of MsSRPN1K.

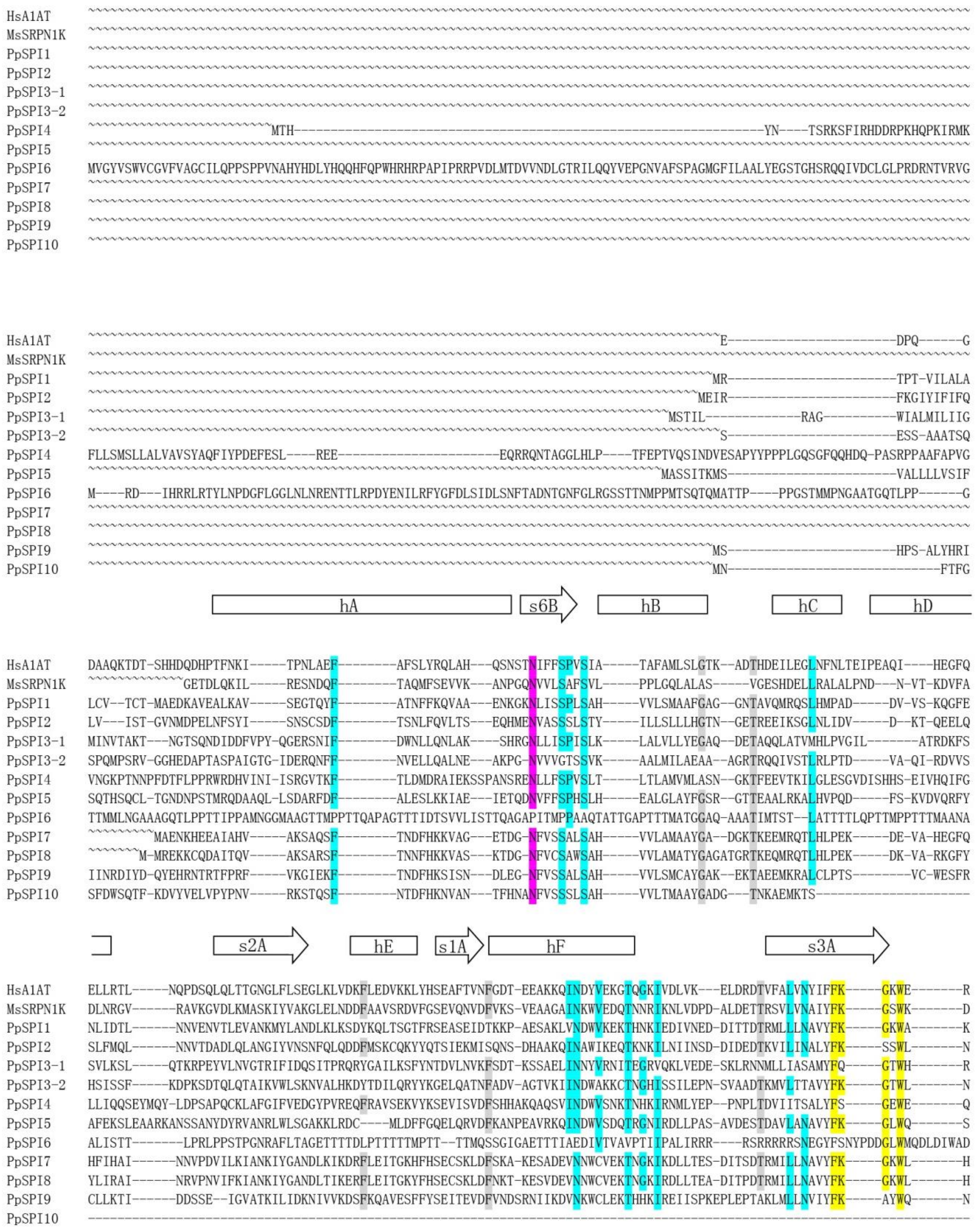
HsA1AT
MsSRPN1K
PpSPI1
PpSPI2
PpSPI3-1
PpSPI3-2
PpSPI4
PpSPI5
PpSPI6
PpSPI7
PpSPI8
PpSPI9
PpSPI10
hA
s6B
hB
hC
hD
s2A
hE
s1A
hF
s3A

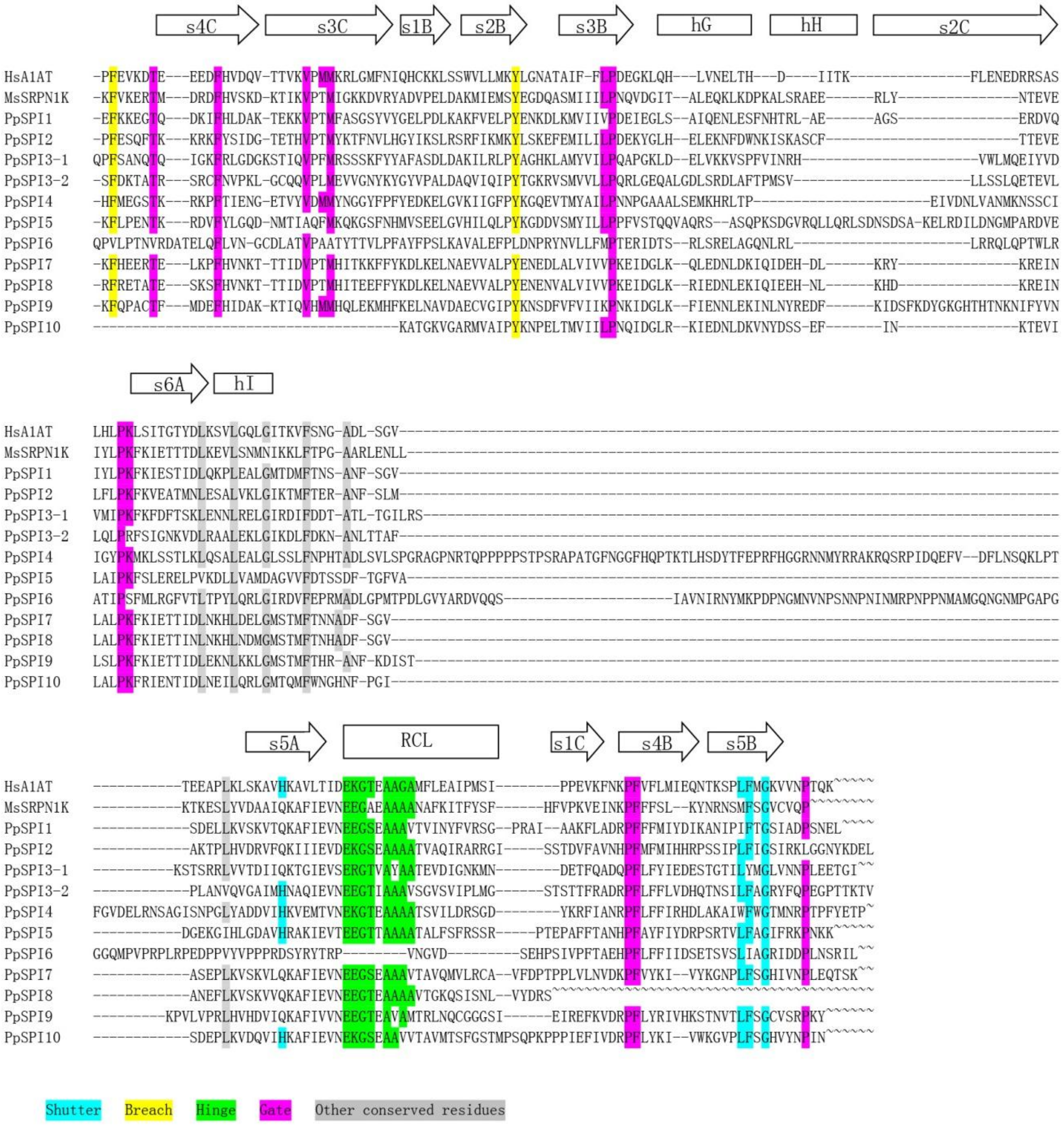
s4C
s3C
s1B
s2B
s3B
hG
hH
s2C
s6A
hI
s5A
RCL
s1C
s4B
s5B
HsA1AT
MsSRPN1K
PpSPI1
PpSPI2
PpSPI3-1
PpSPI3-2
PpSPI4
PpSPI5
PpSPI6
PpSPI7
PpSPI8
PpSPI9
PpSPI10
Shutter
Breach
Hinge
Gate
Other conserved residues

Supplementary Figure S2: Expression levels of SPI genes following different immune challenges. Expression levels of SPI genes following the infection of Gram-negative (*Escherichia coli*),Gram-positive (*Micrococcus luteus*) bacterium or entomopathogenic fungus (*Beauveria bassiana*) were analyzed using qPCR. Time points along the x-axis represent the hours post-infection. Error bars represent the means ±standard deviations from three biological replicates. *P. puparum actin* 1 was used as a housekeeping gene. A two-way ANOVA was used to determine the combined effects of infection and time. The different lowercase letters (a-c) represent the significant difference at the different time points after infection with the same pathogen (p <0.05), and the capital letters (A~C) indicate the significant difference at the same time points after different pathogenic infection (p <0.05).

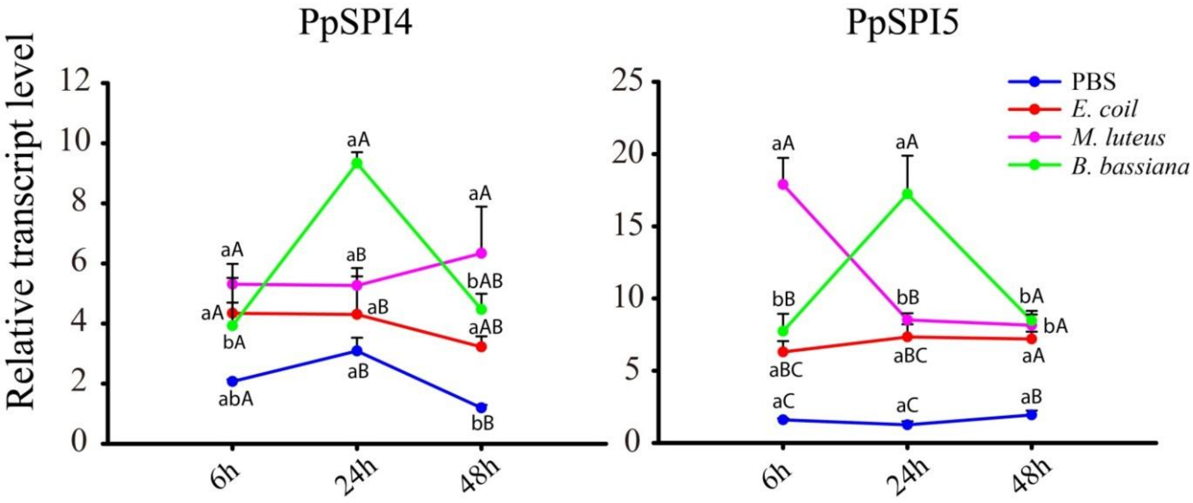

Supplementary Figure S3: Alignment of SPI domains using Clustal X2 and shading using GeneDoc. (A) Kazal family, (B) Pacifastin family, (C) TIL family, (D) Kunitz family, (E) WAP famil

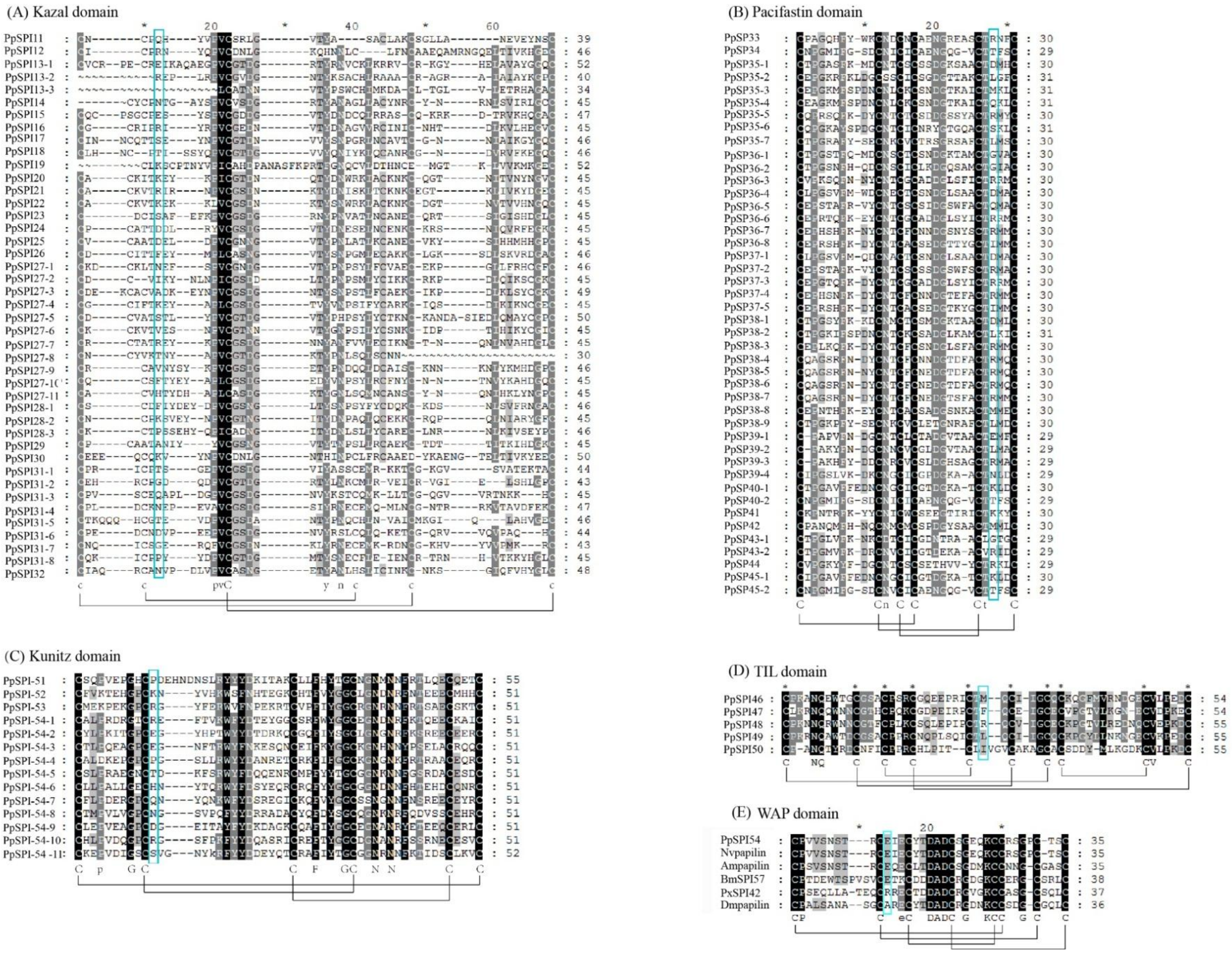
(A) Kazal domain
(B) Pacifastin domain
(C) Kunitz domain
(D) TIL domain
(E) WAP domain

Supplementary Figure S4: The structure domains of *P. puparum* and predicted putative dibasic cleavage sites based on the liable fracture site (RR, RK or KK residues). Green ellipse represents signal peptide and pink square represents Pacifastin domain.

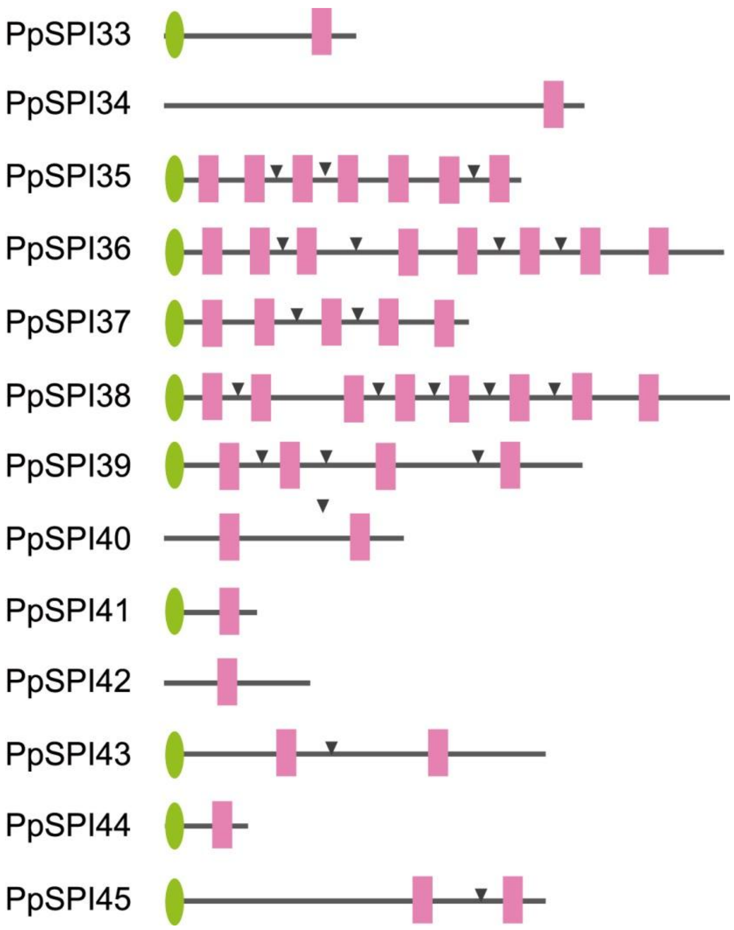

Supplementary Figure S5: Multiple sequence alignment of *P. puparum* A2M-like genes with A2M genes from other species. The signal peptides were marked by red underline. Different color dotted boxs represrnted the A2M-N, A2M-N2, A2M, A2M-COMP and A2M-RECEP domains. The conserved FPETW and GCGEQ sequences were marked with red full line box. The asterisk represented important residue (H) that affects binding specificity of these A2M genes. The GenBank accession number for sequences used in phylogenetic analysis in supplementary Figure S5:AgTep (AAG00600.1, *Anopheles gambiae*); DmTep (NP_523578.1, *Drosophila melanogaster*); HsA2M (P01023.3, *Homo sapiens*); OmA2M (AAN10129.1, *Ornithodoros moubata*).

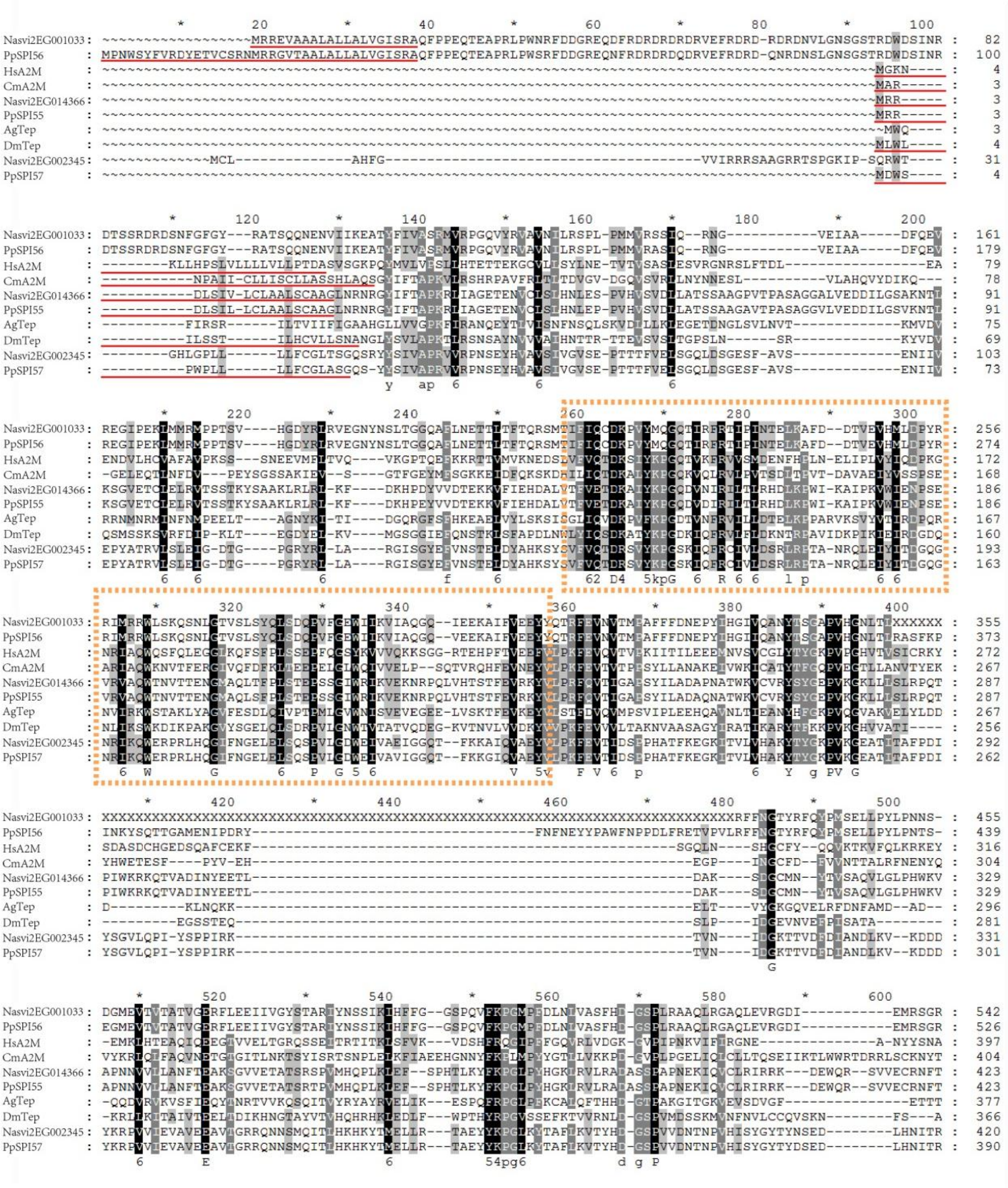

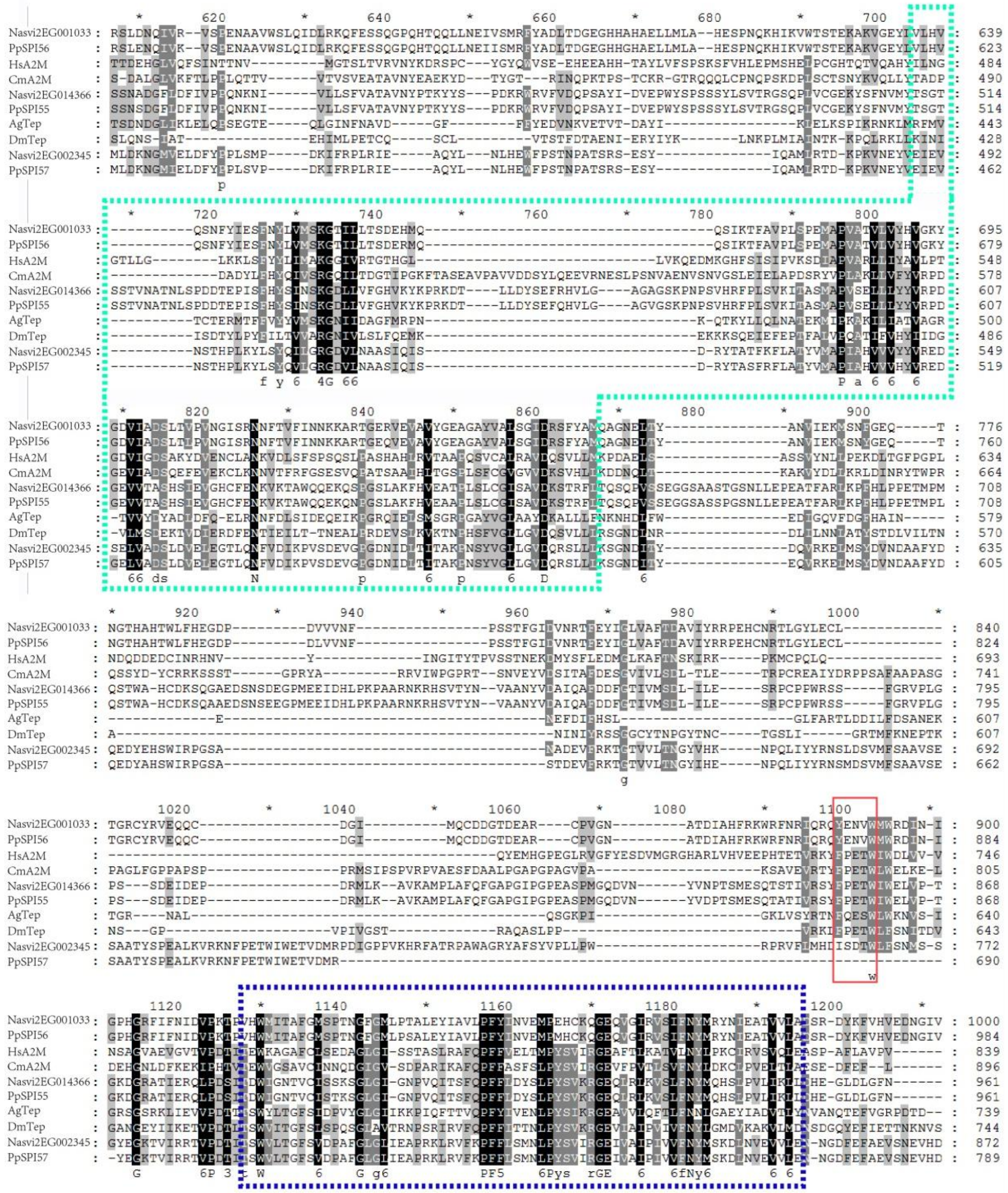
Nasvi2EG001033
PpSPI56
HsA2M
CmA2M
Nasvi2EG014366
PpSPI55
AgTep
DmTep
Nasvi2EG002345
PpSPI57

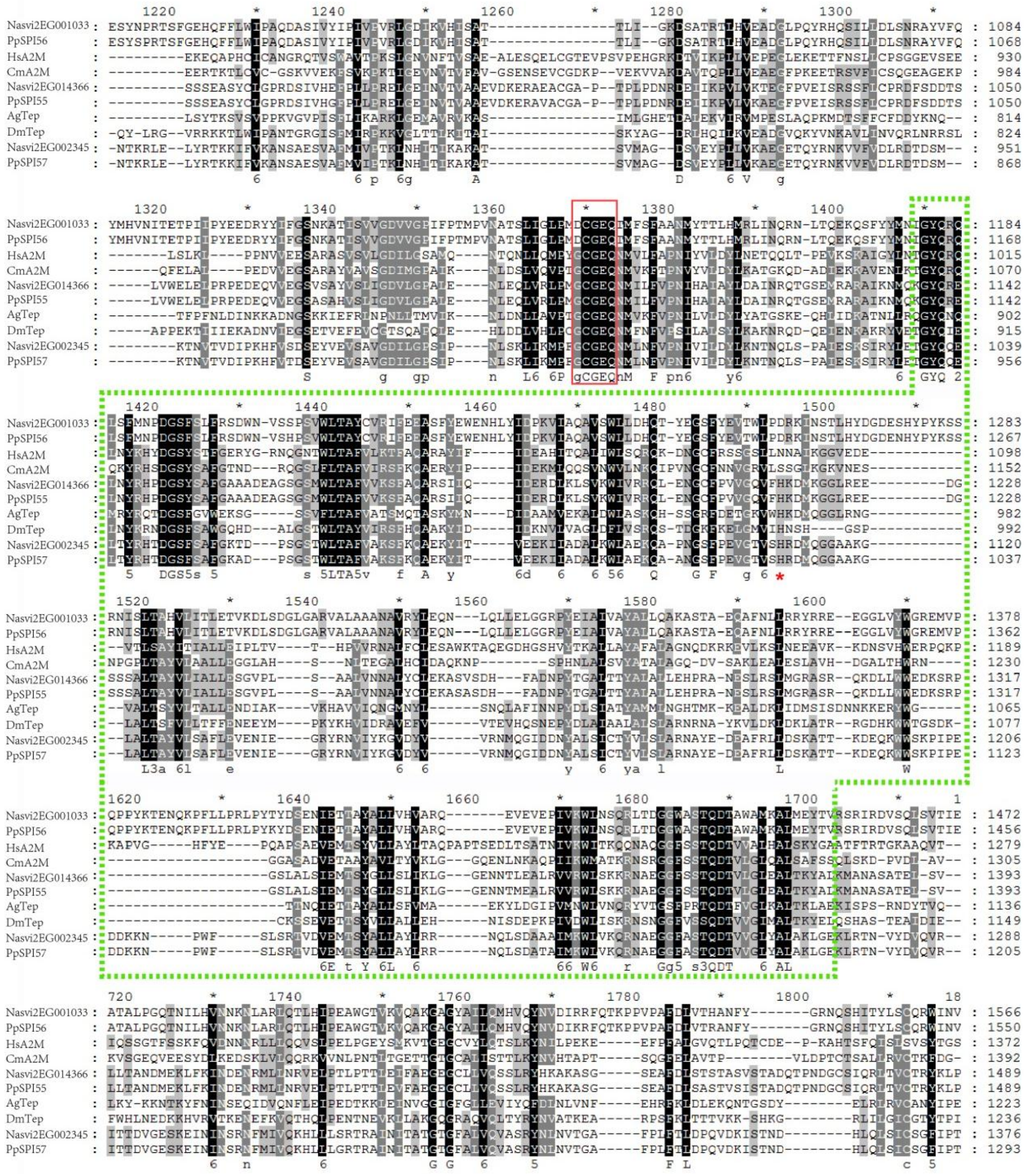

102

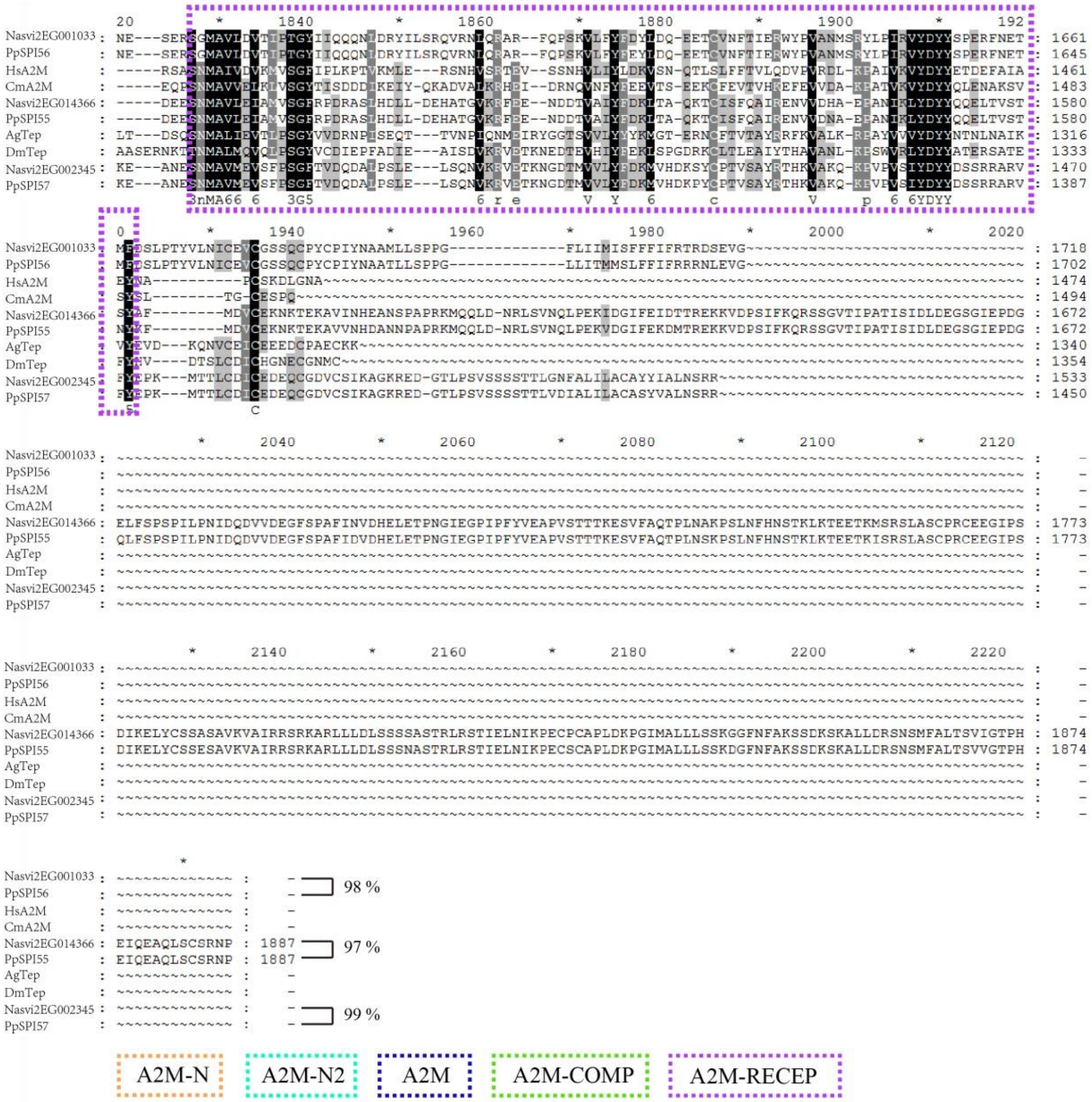

Supplementary Figure S6: Confirmation of developmental stage expressions of SPI genes: Total RNA was extracted from the embryo (E), larvae (L), female pupae (FP), male pupae (MP), female adult (FA) and male adult (MA), and used to analyze the expression patterns of these SPIs using qPCR. *P. puparum* 18s rRNA was used as a housekeeping gene. Error bars represent the means ± standard deviations from three biological replicates. A one-way ANOVA was used to determine the significant difference with different lowercase letter (a-d) (p <0.05).

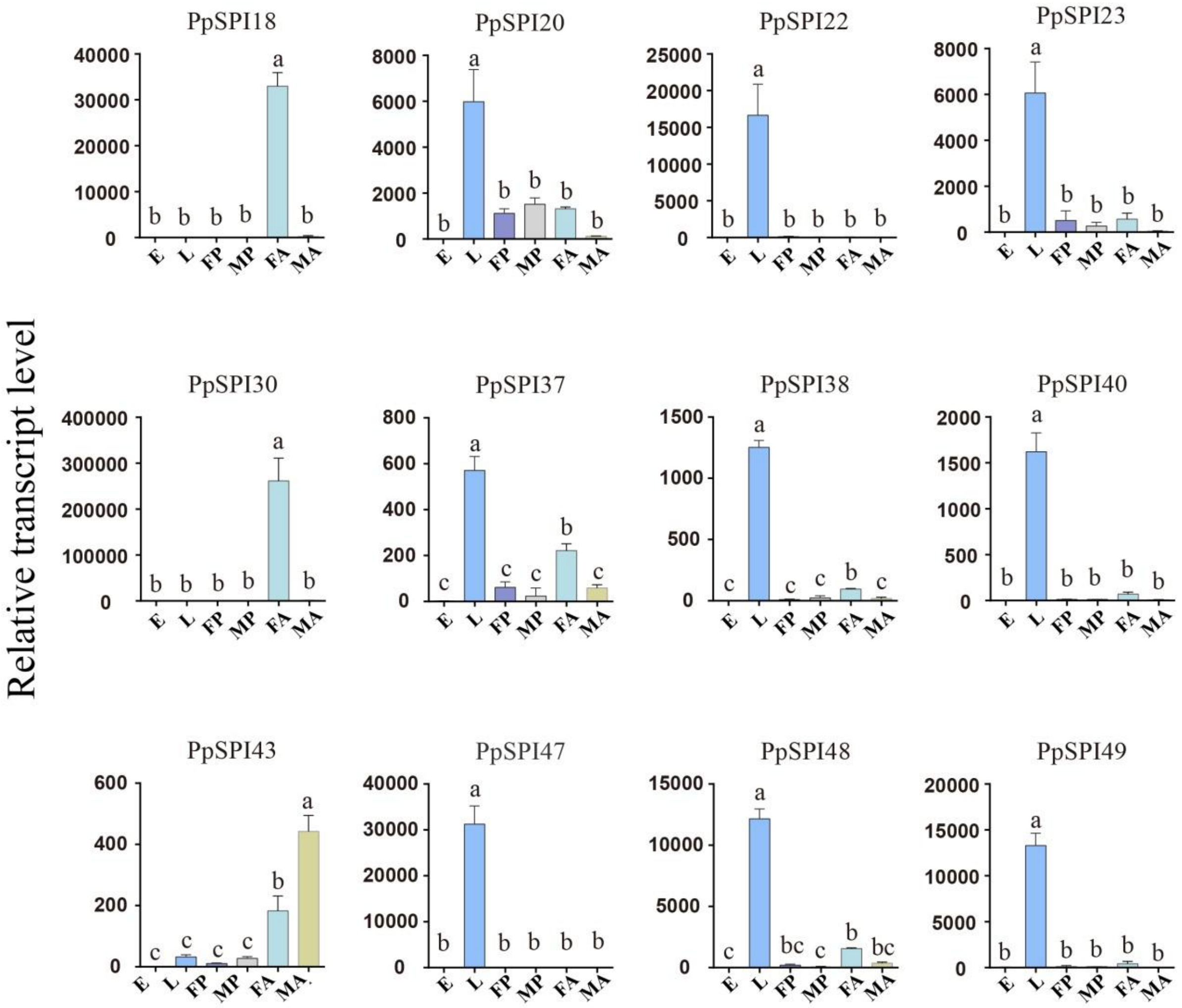
Relative transcript level
PpSPI18
PpSPI20
PpSPI22
PpSPI23
PpSPI30
PpSPI37
PpSPI38
PpSPI40
PpSPI43
PpSPI47
PpSPI48
PpSPI49
E
L
FP
MP
FA
MA
